# A comparative phenotypic and genomic analysis of C57BL/6J and C57BL/6N mouse strains

**DOI:** 10.1186/gb-2013-14-7-r82

**Published:** 2013-07-31

**Authors:** Michelle M Simon, Simon Greenaway, Jacqueline K White, Helmut Fuchs, Valérie Gailus-Durner, Sara Wells, Tania Sorg, Kim Wong, Elodie Bedu, Elizabeth J Cartwright, Romain Dacquin, Sophia Djebali, Jeanne Estabel, Jochen Graw, Neil J Ingham, Ian J Jackson, Andreas Lengeling, Silvia Mandillo, Jacqueline Marvel, Hamid Meziane, Frédéric Preitner, Oliver Puk, Michel Roux, David J Adams, Sarah Atkins, Abdel Ayadi, Lore Becker, Andrew Blake, Debra Brooker, Heather Cater, Marie-France Champy, Roy Combe, Petr Danecek, Armida di Fenza, Hilary Gates, Anna-Karin Gerdin, Elisabetta Golini, John M Hancock, Wolfgang Hans, Sabine M Hölter, Tertius Hough, Pierre Jurdic, Thomas M Keane, Hugh Morgan, Werner Müller, Frauke Neff, George Nicholson, Bastian Pasche, Laura-Anne Roberson, Jan Rozman, Mark Sanderson, Luis Santos, Mohammed Selloum, Carl Shannon, Anne Southwell, Glauco P Tocchini-Valentini, Valerie E Vancollie, Henrik Westerberg, Wolfgang Wurst, Min Zi, Binnaz Yalcin, Ramiro Ramirez-Solis, Karen P Steel, Ann-Marie Mallon, Martin Hrabě de Angelis, Yann Herault, Steve DM Brown

**Affiliations:** 1 Medical Research Council Harwell (Mammalian Genetics Unit and Mary Lyon Centre), Harwell Science Campus, OX11 0RD, UK; 2 The Wellcome Trust Sanger Institute, Wellcome Trust Genome Campus, Hinxton, CB10 1SA, UK; 3 Helmholtz Zentrum München, German Research Centre for Environmental Health, Institute of Experimental Genetics and German Mouse Clinic, Ingolstädter Landstraße 1, Neuherberg, D-85764, Germany; 4 Helmholtz Zentrum München, German Research Centre for Environmental Health, Institute of Developmental Genetics, Ingolstädter Landstraße 1, Neuherberg, D-85764, Germany; 5 Helmholtz Zentrum München, German Research Centre for Environmental Health, Institute of Pathology, Ingolstädter Landstraße 1, Neuherberg, D-85764, Germany; 6 Institut Clinique de la Souris, ICS/MCI, PHENOMIN, GIE CERBM, IGBMC, CNRS, INSERM, 1 Rue Laurent Fries, 67404 Illkirch-Graffenstaden Cedex, France; 7 Faculty of Medical and Human Sciences, University of Manchester, Oxford Road, Manchester, MN13 9PT, UK; 8 Faculty of Life Sciences, University of Manchester, Oxford Road, Manchester, MN13 9PT, UK; 9 AniRA ImmOs phenotyping facility- SFR Biosciences Lyon Gerland- UMS3444/US8, 21 avenue Tony Garnier F-69007 Lyon, France; 10 Medical Research Council Human Genetics Unit, IGMM, University of Edinburgh, Western General Hospital, Crewe Road, Edinburgh, EH4 2XU, UK; 11 Infection and Immunity Division, Roslin Institute, University of Edinburgh, Easter Bush Veterinary Campus, Midlothian, EH25 9RG, UK; 12 Consiglio Nazionale delle Ricerche- Cell Biology and Neurobiology Institute, Via E.Ramarini 32, 00015 Monterotondo Scala, Italy; 13 Department of Infection Genetics, Helmholtz Centre for Infection Research, Inhoffenstraße 7, Braunschweig, 38124, Germany; 14 Mouse Metabolic Facility of the Cardiomet Center, University Hospital, and Center for Integrative Genomics, University of Lausanne, 1015 Lausanne, Switzerland; 15 Center for Integrative Genomics, University of Lausanne, Lausanne, CH-1015, Switzerland; 16 Chair for Developmental Genetics, Technische Universität München, Arcisstr. 21, Munich, 80333, Germany; 17 Max Planck Institute of Psychiatry, Kraepelinstrasse 2, Munich, 80804, Germany; 18 Deutsches Zentrum für Neurodegenerative Erkrankungen, Schillerstrasse 44, Munich, 80336, Germany

**Keywords:** Mouse inbred lines, sequence variation, mouse phenotyping, gene knockout, C57BL/6

## Abstract

**Background:**

The mouse inbred line C57BL/6J is widely used in mouse genetics and its genome has been incorporated into many genetic reference populations. More recently large initiatives such as the International Knockout Mouse Consortium (IKMC) are using the C57BL/6N mouse strain to generate null alleles for all mouse genes. Hence both strains are now widely used in mouse genetics studies. Here we perform a comprehensive genomic and phenotypic analysis of the two strains to identify differences that may influence their underlying genetic mechanisms.

**Results:**

We undertake genome sequence comparisons of C57BL/6J and C57BL/6N to identify SNPs, indels and structural variants, with a focus on identifying all coding variants. We annotate 34 SNPs and 2 indels that distinguish C57BL/6J and C57BL/6N coding sequences, as well as 15 structural variants that overlap a gene. In parallel we assess the comparative phenotypes of the two inbred lines utilizing the EMPReSSslim phenotyping pipeline, a broad based assessment encompassing diverse biological systems. We perform additional secondary phenotyping assessments to explore other phenotype domains and to elaborate phenotype differences identified in the primary assessment. We uncover significant phenotypic differences between the two lines, replicated across multiple centers, in a number of physiological, biochemical and behavioral systems.

**Conclusions:**

Comparison of C57BL/6J and C57BL/6N demonstrates a range of phenotypic differences that have the potential to impact upon penetrance and expressivity of mutational effects in these strains. Moreover, the sequence variants we identify provide a set of candidate genes for the phenotypic differences observed between the two strains.

## Background

The development of a comprehensive mouse embryonic stem cell (ESC) mutant resource by the International Knockout Mouse Consortium (IKMC) [1] is a crucial step in the systematic functional annotation of the mouse genome. To date, ESC mutant lines are available for around 15,000 mouse genes, providing a very significant resource for the generation of mutant mice and their subsequent phenotypic analysis. The IKMC resource is being used by the International Mouse Phenotyping Consortium (IMPC), which plans over the next 5 years to generate and carry out broad-based phenotyping on 5.000 mouse mutant lines as the first step towards a comprehensive encyclopedia of mammalian gene function [2].

All IKMC mutant clones have been generated using a C57BL/6N ESC line [1]. Moreover, chimaeras generated from IKMC clones as part of the IMPC program have been bred to C57BL/6N mice, thus maintaining the mutations on an isogenic background. The use of C57BL/6N for these major functional genomics programs brings into perspective the genetic relationship between the C57BL/6N strain and other inbred strains that have been the focus of mouse genetics research in the past. In particular, a considerable number of mouse genetic resources have been developed using the C57BL/6J strain, including a variety of reference populations such as recombinant inbred lines [3,4], consomics [5], heterogeneous stocks [6] and the Collaborative Cross [7]. Moreover, a large number of spontaneous mutations have been identified on the C57BL/6J background. As a consequence, the C57BL/6J line was the natural choice to provide the first reference sequence of the mouse genome [8,9]. The significant usage of both the N and J sub-strains throughout the wider biomedical science communities emphasizes the need to understand better the genetic and phenotypic relationships between these two inbred strains, and how they might affect our understanding of genetic mechanisms and phenotype outcomes.

The inbred C57BL/6 mouse strain was established at the Jackson Laboratory from the parental strain C57BL at F24 in 1948. In 1951, at F32, it was then passed on to the National Institutes of Health (NIH), leading to the C57BL/6N line. The C57BL/6NTac sub-strain was established at F151, following the transfer of the C57BL/6N line to Taconic Farms in 1991 [10]. Thus, at the current time, C57BL/6J and C57BL/6N have been separated for around 220 generations. Early assessment of the genetic variation between the C57BL/6J and C57BL/6N sub-strains using a panel of 1,427 single-nucleotide polymorphism (SNP) loci identified only 12 SNPs (0.8%) between the two strains [10], reflecting their close genetic relationship.

In 2011, an extensive analysis of genomic variation in 17 inbred strains catalogued an extraordinarily large number of variants, including 56.7 M SNPs, 8.8 M small indels and 0.28 M structural variants (SVs) across both the classic laboratory strains and the wild type-derived lines [11]. In addition, these analyses illustrated the potential to relate sequence variation to aspects of phenotypic variation between mouse strains. Importantly, the analyses provided an insight into the molecular and genetic basis of quantitative traits that distinguish the phenotypic characteristics of inbred strains [11]. Small-effect quantitative trait loci (QTL) were found to be more often due to intergenic variation, and are unlikely to be the result of structural variation. By contrast, larger-effect QTL are usually explained by intronic variation. However, for the small proportion of QTL of very large phenotypic effect, there is a significant enrichment of coding variation, with an increasing frequency of SVs and small indels. Although overall, the proportion of SVs within the mouse genome causing major phenotypic effects is small, it is likely that SVs that cause phenotypic change will provide significant insights into gene function [11]. This work emphasizes the utility and importance of cataloguing genomic variation in the mouse and analyzing its contribution to phenotypic effects.

In this paper, we focus our analysis on a detailed genomic and phenotypic comparison of the C57BL/6N and C57BL/6J strains, aiming to relate the underlying genomic differences to phenotypic outcome. We expanded and refined the analysis of the genome sequences of the two inbred strains. Importantly, using the new short-read genome sequence of the C57BL/6J generated by the Broad Institute and improved analytical tools, we identified a high-quality set of variants including SNPs, small indels, and SVs that distinguish the C57BL/6N and C57BL/6J strains, with a particular focus on cataloguing variation in coding sequences. Using a combination of experimental methods, we validated all coding variants and SVs generating a significantly higher quality variant dataset than that generated from the 17 Mouse Genomes Project, with a null false-positive rate. In parallel, we undertook a comprehensive phenotypic comparison and examined the relationship between genome variation and phenotypic changes in these two sub-strains.

## Results

### Genome sequence comparisons of C57BL/6N and C57BL/6J mice for SNPs and small indels

We utilized paired-end alignment of C57BL/6N to the reference genome (C57BL/6J) from the 17 Mouse Genomes Project [10]. However, the list of differentiating variants (SNPs, small indels, and SVs) between the two genomes was newly created using novel inbuilt procedures in order to increase the likelihood of identifying accurate putative sequence changes. A key analysis step in identifying a high-quality set of variants from the alignment was to utilize the newly generated short-read genome sequence of C57BL/6J generated by the Broad Institute. This enabled us to identify assembly errors in the reference sequence. In addition, we updated the variant detection method: first, by using different and/or more evolved software to detect variants; second, by performing manual curation on all coding variants, and third, by extensive validation of a large proportion of the variants (including all coding variants) to confirm the sequence predictions. These steps provided a robust dataset of high-quality coding variants, considerably reducing the false-positive rate.

To identify SNPs and small indels differentiating the C57BL/6J and C57BL/6N strains, we used the paired-end reads generated from the 17 Mouse Genomes Project [11]. We called variants using the Genome Analysis Toolkit (GATK) [12], and found 681,220 variants that distinguish the C57BL/6J and C57BL/6N strains. Using the short-read genome sequence of C57BL/6J generated by the Broad Institute [13], we were able to filter out prospective sequencing errors by removing variants common to the Broad C57BL/6J sequence, thus counteracting discrepancies in the reference while improving the false-negative rate. The remaining reads were filtered with an allele ratio of less than 0.8 (heterozygous) and covered by less than 3 or greater than 150 reads. These steps significantly reduced the list, resulting in 10,794 putative variants that were subjected to further analyses.

Using Sequenom, PyroSequencing, and Sanger sequencing, we validated all coding variants and a subset of the non-coding variants, which included 762 SNPs and 169 small indels. Assays were carried out using a panel of four C57BL/6J and four C57BL/6N samples in order to confirm genotypes (see Materials and methods). We considered a variant to be validated when all four C57BL/6J and C57BL/6N samples showed consistent genotypes within a sub-strain and variants between the sub-strains. During the validation process, we eliminated 363 variants for a number of reasons, including heterozygous and inconsistent genotypes and PCR failures. For the remaining 568, 236 were confirmed as variant between the sub-strains (see Additional file 1, Table S1).

Using the annotation programs NGS-SNP and Annovar [14,15], the genomic location and other gene features were examined. The final validated sequence variants between C57BL/6J and C57BL/6N consisted of 34 coding SNPs, 2 coding small indels, 146 non-coding SNPs, and 54 non-coding small indels. Coding variants included 32 missense SNPs, 1 nonsense mutation, 1 splicing mutation, and 2 frameshift mutations (Table 1). We found that all variants except one (*Zp2*, chromosome 7) were private to either C57BL/6J or C57BL/6N, and were not found in any of the 16 other inbred strains recently sequenced [11].

**Table 1:** Coding single-nucleotide polymorphisms and small indels identified in the comparison between C57BL/6N and C57BL/6J

Chr	Position	B6J base	B6N base	Strain	Gene name	B6J amino acid	B6N amino acid
Nonsense polymorphism							
13	65023280	C	T	B6N	*Spata31*	Arginine	*(stop)
Missense polymorphisms							
1	59904011	G	A	B6N	*Bmpr2*	Arginine	Glutamine
3	95538799	T	C	B6J	*Ecm1*	Isoleucine	Valine
3	96658480	A	G	B6J	*Pdzk1*	Asparagine	Aspartic acid
4	21800831	C	G	B6J	*Sfrs18*	Arginine	Glycine
4	137777588	C	T	B6N	*Hp1bp3*	Leucine	Phenylalanine
4	140354038	A	G	B6N	*Padi3*	Leucine	Proline
4	148318468	T	C	B6J	*Casz1*	Leucine	Proline
5	90204376	C	T	B6N	*Adamts3*	Valine	Isoleucine
5	97187161	T	C	B6J	*Fras1*	Leucine	Proline
5	113191741	C	T	B6N	*Myo18b*	Arginine	Histidine
6	39350455	T	A	B6J	*Mkrn1*	Asparagine	Tyrosine
7	3222538	T	C	B6J	*Nlrp12*	Lysine	Arginine
7	63386662	G	A	B6J	*Herc2*	Glycine	Aspartic acid
7	86256240	A	C	B6J	*Acan*	Histidine	Proline
7	110121823	C	T	B6N	*Olfr577*	Valine	Isoleucine
7	127278693	G	A	B6N+Spretus	*Zp2*	Alanine	Valine
7	129311164	C	T	B6N	*Plk1*	Arginine	Tryptophan
9	24935069	C	G	B6N	*Herpud2*	Valine	Leucine
10	66700922	T	C	B6J	*Jmjd1c*	Leucine	Proline
10	78632222	A	G	B6N	*Vmn2r80*	Asparagine	Serine
10	87554578	T	C	B6N	*Pmch*	Isoleucine	Threonine
11	46036117	G	A	B6N	*Cyfip2*	Serine	Phenylalanine
11	90341985	C	T	B6N	*Stxb4*	*Stxb4*	Threonine
13	21560172	A	G	B6J	*Nkapl*	Glycine	Arginine
13	73465884	A	G	B6J	*Ndufs6*	Valine	Alanine
13	93833534	C	G	B6J	*Cmya5*	Alanine	Proline
14	70986011	G	T	B6N	*Fam160b2*	Serine	Arginine
15	11266138	G	T	B6N	*Adamts12*	Cysteine	Phenylalanine
15	77468437	A	C	B6J	*Apol11b*	Isoleucine	Arginine
16	35291630	G	A	B6N	*Adcy5*	Valine	Methionine
17	47537359	T	C	B6J	*Guca1a*	Isoleucine	Valine
X	131227581	C	A	B6N	*Armcx4*	Alanine	Aspartic acid
Splice site polymorphism							
5	54280548	A	G	B6J	*Tbc1d19*	-	-
Frameshift 1 bp deletions							
1	141133664	G	-	B6N	*Crb1*	-	-
9	65127938	G	-	B6J	*Cilp*	-	-

Abbreviations: Chr, chromosome.

### Genome sequence comparisons of C57BL/6N and C57BL/6J mice for structural variants

Again, employing the paired-end reads generated from the 17 Mouse Genomes Project [11] and a combination of four computational methods [16], we identified 551 SVs between C57BL/6J and C57BL/6N. As described elsewhere [17], we visually inspected short-read paired-end mapping at these 551 SV sites in the 17 sequenced inbred strains of mice [11] and in the Broad J sequenced genome [13]. By doing this, we were able to retain 81 of the 551 sites for further experimental analyses (470 predicted sites were found to be false because of paired-end mapping errors). PCR and Sanger-based sequencing analyses at these 81 retained sites allowed us to remove a further 38 sites, which were confirmed to non-polymorphic between C57BL/6J and C57BL/6N because of reference errors. Finally, all 43 predicted variants were validated as authentic SVs differentiating the C57BL/6J and C57BL/6N strains (Table 2), resulting in a null false-positive rate.

**Table 2:** Structural variants (SVs) between C57BL/6N and C57BL/6J

Chr	SV start^a^	SV stop^a^	Ancestral event	Strain	Gene	Overlap
1	149518394	149524878	LINE Ins	B6J	-	-
2	7325700	7330977	IAP Ins	B6J	-	-
2	70619835	70620080	SINE Ins	B6J	*Tlk1*	Intron
3	77975065	77977953	Del	B6N	-	-
3	5049018	5055845	LINE Ins	B6J	-	-
3	60336036	60336037	Del (large)	B6J	*Mbnl1*	Intron
3	41885819	41887255	LINE Ins	B6J	-	-
3	18484710	18484889	Del	B6N	-	-
4	101954274	101954395	Del	B6N	*Pde4b*	Intron
4	116051393	116051799	MaLR Ins	B6J	*Mast2*	Intron
5	46376307	46377852	LINE Ins	B6J	-	-
5	90356490	90356491	Del (~300 bp)	B6J	-	-
5	146248861	146261885	Ins	B6J+others	-	-
6	18112291	18119019	LINE Ins	B6J	-	-
6	62964974	62972907	LINE Ins	B6J	-	-
6	86478779	86479400	Ins	B6J	-	-
6	103669536	103676487	LINE Ins	B6J	*Chl1*	Intron
6	104207081	104214434	LINE Ins	B6J	-	-
7	92095990	92096149	Del	B6N	*Vmn2r65*	Exon
7	27636128	27748456	Ins	B6J	*Cyp2a22*	Entire
7	100892501	100899058	LINE Ins	B6J	-	-
7	139306094	139307981	MaLR Ins	B6J	*Cpxm2*	Intron
8	16716381	16716382	Del (large)	B6J	*Csmd1*	Intron
9	25674550	25674770	SINE Ins	B6J	-	-
9	58544415	58546304	MaLR Ins	B6J	*2410076I21Rik*	Intron
10	3039196	3039197	Del (large)	B6J	-	-
10	29339441	29345955	LINE Ins	B6J	-	-
10	32536420	32543464	LINE Ins	B6J	*Nkain2*	Intron
10	49543303	49550645	LINE Ins	B6J	-	-
11	104906390	104906621	Del	B6N	-	-
11	119560391	119566827	MTA Ins	B6J	*Rptor*	Intron
12	42023964	42032747	Del	B6N	*Immp2l*	Intron
13	71224557	71231011	MTA Ins	B6J	-	-
13	120164268	120164269	Del (large)	B6J	*Nnt*	Exon
14	112825585	112832341	LINE Ins	B6J	-	-
15	49554596	49554597	Ins (large)	B6N	-	-
15	31106173	31106382	VNTR	-	-	-
16	6115804	6138105	Del	B6N	-	-
17	60286367	60286368	Ins (~2000 bp)	B6N	-	-
18	4809271	4809272	Del (~1200 bp)	B6J	-	-
19	12863187	12863188	Del (~1800 bp)	B6J	*Zfp91*	Intron
X	15697909	15697910	Del (~400 bp)	B6J	-	-
X	95155499	95163160	LINE Ins	B6J	-	-

Abbreviations: Del, deletion; IAP, intracisternal A particle; Ins, insertion; LINE, long interspersed nuclear element; MaLR, mammalian apparent long terminal repeat retrotransposon; MTA, member of transcript retrotransposon; SINE, short interspersed nuclear element; VNTR, variable number tandem repeat.

**^a^**Start and stop coordinates are given for MGSCv37 of the mouse reference genome.

Of the 43 SVs, 15 overlap with a gene (Table 2), including 12 variants that lie within non-coding regions of genes, 2 variants that affect the coding region of the gene (*Vmn2r65 *(Vomeronasal 2, receptor 65) and *Nnt *(nicotinamide nucleotide transhydrogenase)), and 1 that affects the entire gene *Cyp2a22 *(cytochrome P450, family 2, subfamily a, polypeptide 22). Only 1 of the 15 variants is known and has been already associated with a phenotype, *Nnt *[18]; the remaining 14 are novel, and for several we discuss their potential biological functions below.

Using the rat as an outgroup species, we next inferred the origin of the 43 SVs between C57BL/6J and C57BL/6N, and found that 27 variants were the product of retrotransposition, 15 were non-repeat-mediated SVs, and 1 was a variable number tandem repeat (VNTR) (Table 2). Remarkably, almost all variants were private to either C57BL/6J or C57BL/6N (Table 2).

### Comprehensive phenotypic assessment of C57BL/6N and C57BL/6J mice

In parallel to the genomic analyses, the European Mouse Disease Clinic (EUMODIC) consortium has carried out a comprehensive phenotypic comparison of the C57BL/6NTac and C57BL/6J strains. EUMODIC comprises four mouse centers [19] carrying out broad-based primary phenotyping of 500 mouse mutant knockout lines generated from the European Conditional Mouse Mutagenesis (EUCOMM) and Knockout Mouse (KOMP) projects within the IKMC program. Cohorts of mice from each mutant line enter the European Mouse Phenotyping Resource of Standardised Screens slim (EMPReSSslim) phenotype assessment, which consists of two phenotyping pipelines, together comprising 20 phenotyping platforms (identified by an ESLIM__procedure_number) that are carried out from 9 to 15 weeks (see Additional file 2, Figure S1). The methods for performing each screen are detailed in the standard operating procedures (SOPs) that can be found in the EMPReSS database [20]. Data were acquired on 413 phenotype parameters along with 146 metadata parameters, and entered into the EuroPhenome database [19]. As part of this work, we have been capturing extensive control data on the baseline phenotype of C57BL/6NTac. We have also taken this opportunity to investigate the phenotype of C57BL/6J mice and to compare this with C57BL/6NTac (henceforth referred to as J and N respectively).

For each line, N and J, age-matched mice have been analyzed through both EMPReSSslim pipelines. Data were acquired from all four centers in the consortium for 19 of the 20 platforms from the pipeline, excluding fluorescence-activated cell sorting (FACS) analyses (see Additional file 2, Figure S1). The EMPReSSslim protocols have been rigorously standardized in the EUMODIC consortium; however, there remain some differences in, for example, equipment and diet, and this is captured in the metadata sets within EuroPhenome. There will of course be other unrecognized environmental differences between centers. Collectively, these may contribute to gene-environment differences and phenotype outcome, but we did not seek to systematically define these effects, instead focusing on phenotypes that are concordant between centers and are clearly robust to unrecognized environmental perturbations. Data from the N and J cohorts from each center were deposited in EuroPhenome and have been subjected to a statistical analysis for each center (see Materials and methods). It is important to note here that comparisons between N and J were performed within, not between centers. Statistical analysis of results between centers was not performed, as experiments could not be completely controlled between centers because of environmental and other variables and differences in numbers of animals analyzed in each center (see Additional file 3, Figure S2a-d). We thus chose to adopt an approach that focused on strain comparisons within individual centers as opposed to generating a multi-center statistical model that examined an overall statistical difference between the two strains. However, replication of the N and J comparison across multiple centers provided us with additional power in substantiating significant phenotypic differences between the two strains [see Materials and methods]. In addition to the analysis of N and J through the EMPReSSslim primary phenotyping pipeline at the four centers, other partners within the EUMODIC consortium have applied a wider range of often more sophisticated phenotyping tests to gather additional information, some of which explores further and aims to substantiate the phenotypic differences revealed through EMPReSSslim.

**Figure 1:**
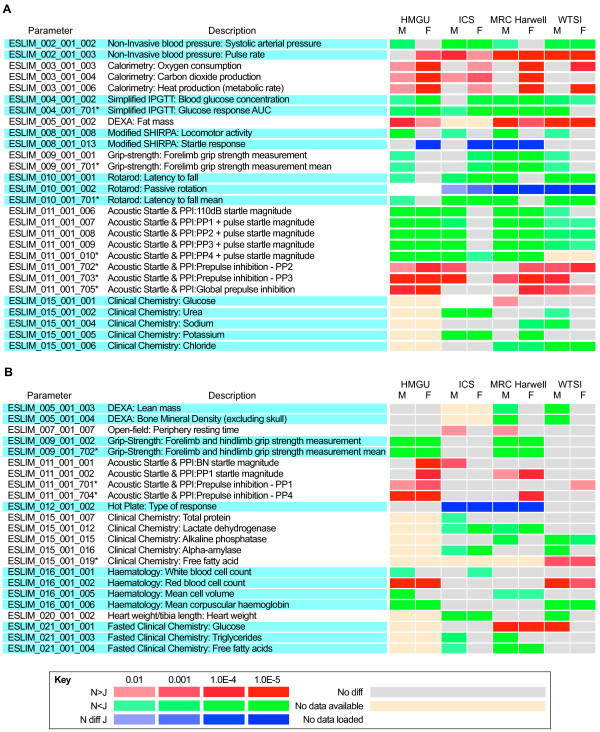
**Heat maps illustrating significant differences in phenotype parameters between C57BL/6N and C57BL/6J male and female mice**. Parameters were assessed from each of the four centers: Helmholtz Zentrum Munchen (HMGU), Institut Clinique Souris (ICS), MRC Harwell, and the Wellcome Trust Sanger Institute (WTSI). Parameter designations and parameter descriptions are from EMPReSSslim [37]. Significance levels and the direction of the effect (red and green) are defined in the key. Significant differences for categorical data are illustrated in blue. **(A, B) **Phenotype parameters showing a significant difference between N and J in **(A) **three or more centers, and **(B) **in two centers but no evidence of trends in the other centers.

**Figure 2:**
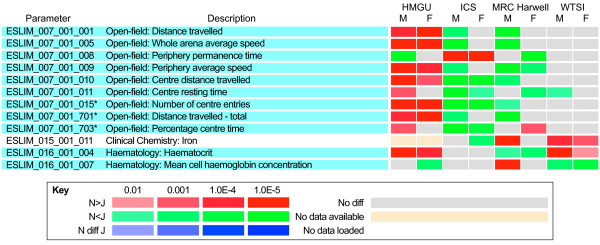
**Heat maps illustrating significant differences in phenotype parameters between C57BL/6N and C57BL/6J male and female mice**. Parameters were assessed from each of the four centers (Helmholtz Zentrum Munchen (HMGU), Institut Clinique Souris (ICS), MRC Harwell, and the Wellcome Trust Sanger Institute (WTSI)). Phenotype parameters that showed significant differences in two or more centers, but the opposite trend in another center. Parameter designations and parameter descriptions are from EMPReSSslim. Significance levels and the direction of the effect (red and green) are defined in the key.

In analyzing the data, we focused first on identifying phenotype parameters that showed a consistent and significant difference between N and J in three or more centers. We identified 27 phenotype parameters in this class (Figure 1a; see Additional file 3, Figure S2a, e). In several cases, these differences were supported by data from secondary analysis, and we discuss these instances below. We also uncovered a second class of parameters for which similar trends were seen in two centers, but no evidence of trends was seen in the other two centers (Figure 1b; see Additional file 3, Figure S2b, f). However, our statistical analysis (see Materials and methods) indicates that for this class of parameters the overall significance of N versus J differences is low, and the trends observed should be treated with caution. In several of these cases however, the observed trends are consistent with phenotypes found in the first class of parameters. We also identified a third but small class of parameters that showed highly significant differences in two or more centers (Figure 2; see Additional file 3, Figure S2d, h), but unexpectedly, the opposite trend in one of the centers. We discuss the reasons for these anomalies, which in some cases presumably arise from gene-center interactions. The final class represents a large number of tests in which we did not observe any consistent and significant differences across the centers, concluding that these are more likely to be false positives rather than evidence for N/J differences (see Additional file 3, Figure S2c, g).

#### Dysmorphology and ophthalmology

**Figure 3:**
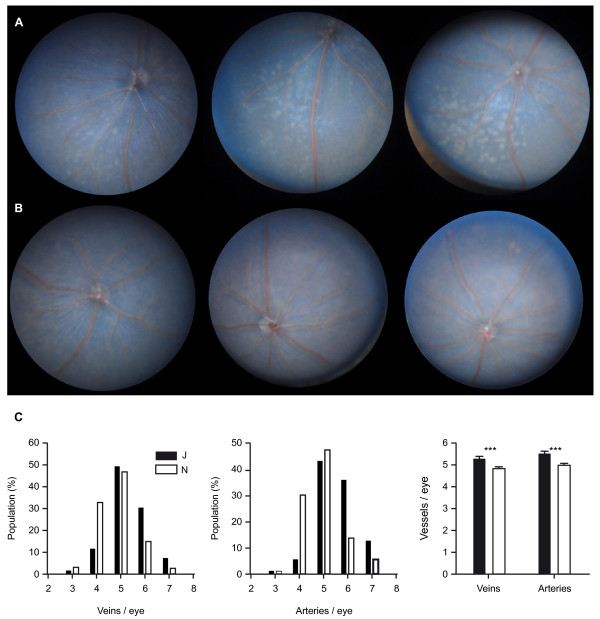
**Morphological and functional differences between C57BL/6N and C57BL/6J mice eyes**. **(A) **Whereas white flecks were absent from C57BL/6J fundus, they were frequently detected in the ventral retina from C57BL/6N mice, with various degrees of severity, as illustrated here by three C57BL/6N fundus images. Depending on the investigating center, C57BL/6N mice with at least one eye affected represented 69.2% (*n *= 70 males + 34 females; ICS), 44.6% (*n *= 145 males + 158 females; GMC), or 23.0% (*n *= 184 males + 194 females; WTSI) of the population, whereas no flecking was detected in C57BL/6J mice (ICS: 29 males; GMC: 75 males + 75 females; WTSI: 34 males + 28 females). **(B) **Both vein and artery numbers differed from mouse to mouse in both strains, usually being between (left) three and (right) seven, with (middle) a mean of around five, as can be seen in three fundus images from C57BL/6J mice. **(C) **Quantification of veins and arteries in male C57BL/6N and C57BL/6J mice (*n *= 140 and *n *= 70 eyes, respectively). The mean number of veins per eye was 4.8 ± 0.1 for C57BL/6N (*n *= 122 eyes) versus 5.3 ± 0.1 for C57BL/6J mice (*n *= 138 eyes). Both differences were significant (*P *< 0.001, Mann-Whitney rank sum test).

We found no evidence for any major differences in morphological features between N and J, including X-ray analysis of the skeleton. However, a number of ophthalmological differences between the two strains were identified. Analysis of the general visual functions using the virtual optokinetic drum [21] found reduced vision in N compared with J mice (N: 0.314 cycle/degree, 95% CI 0.305 to 0.323, *n *= 89; J: 0.399 cycles/degree, 95% CI 0.394 to 0.404, *n *= 128; p < 0.001, Student's *t*-test). This did not reflect differences in lens opacities, as quantitative analysis using a Scheimpflug camera found transparent lenses in both strains (N: 5.2 + 0.5%, *n *= 10; J: 5.0% + 0.5% opacity, *n *= 10). White flecks were seen in the fundus of N mice at a high frequency, which were absent in J mice (Figure 3A). This is probably due to the presence of the *Crb1*^*rd8 *^mutation in N mice, as reported previously [22], although in our case the flecks were seen only in the ventral retina, with variations in fleck size and affected area between mice (Figure 3A). Further studies using topical fundus endoscopy [23] showed that the number of main vessels was variable, ranging between three and seven for veins and three and eight for arteries (Figure 3B), and a given mouse could have non-matching numbers between the two eyes. The mean number of both veins and arteries was significantly higher (*P *< 0.001) in J than in N mice (Figure 3C).

#### Cardiovascular

Non-invasive blood pressure measurements (ESLIM_002) showed that systolic arterial pressure was significantly higher in J than in N mice, although the significance of the effect was found to be variable between sexes and between centers. Moreover, all centers observed that pulse rate was significantly higher in N than in J mice. However a secondary partner within the consortium found that heart rate under anesthetic was significantly lower in N than in J male mice, reflected in a long inter-beat (RR) and QTc interval. We also found that heart weight normalized to tibia length (ESLIM_020) was significantly lower in N than in J mice in two of the centers, and these results were independently confirmed by secondary analysis. Further studies of cardiac structure and function by echocardiography and of cardiac contractile function by hemodynamics failed to reveal any differences between N and J (data not shown).

#### Metabolism

For indirect calorimetry measurements of free-fed mice (ESLIM_003), we found a consistent difference between N and J for O_2 _consumption, CO_2 _production, and heat production. J mice showed reduced gas exchange and lower energy expenditure (heat production or metabolic rate) compared with N, which was generally more marked in females. In secondary phenotyping with fasted indirect calorimetry, there was a trend towards lower energy expenditure in J versus N during the night period. This was possibly associated with decreased ambulatory activity in J and lower food intake in J compared with N during the night period, especially on re-feeding (data not shown). There was no consistent difference in activity in the free-fed calorimetry screen (ESLIM_003) in the two centers where activity was measured (see Additional file 3, Figure S2c, g). Simplified intraperitoneal glucose tolerance tests (IPGTTs) (ESLIM_004) showed impaired glucose tolerance in J versus N mice. These observations on glucose metabolism are consistent with the known deletion of the *Nnt *gene specific to J mice [18], which has been shown to play a role in the regulation of the insulin response in pancreatic beta cells.

**Figure 4:**
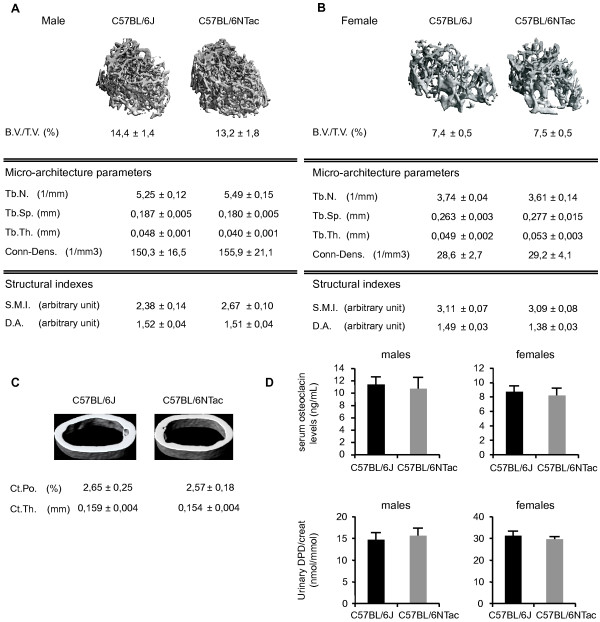
**Micro-computed tomography (μCT) analysis of distal femur showed similar trabecular bone parameters in 14-week-old C57BL/6J and C57BL/6N mice**. **(A) **Males and **(B) **females. **(C) **Cortical bone parameters from midshaft femur of 14-week-old male mice were also unchanged between the two strains. **(D) **Measurement of serum osteocalcin and urinary deoxypyridinoline (bone formation and bone resorption markers, respectively), indicates that bone turnover was identical between 14-week-old C57BL/6J and C57BL/6N. Abbreviations: BV/TV, bone volume/tissue volume; TbN, trabecular number; TbSp, Trabecular spacing; Conn-Dens, Connectivity density; SMI, structural model index (0 for parallel plates, 3 for cylindrical rods); DA, degree of anisotropy; CtPo, cortical porosity; CtTh, cortical thickness; DPD, deoxypyridinoline; creat, creatinin.

Dual energy X-ray absorptiometry (DEXA) body composition and bone densitometry measurements (ESLIM_005) showed that N mice have increased fat mass (both absolute and normalized to weight). Furthermore, DEXA measurements indicated that J mice have increased lean mass compared with N. In two of the centers, bone mineral density measurements were higher in J male mice; however, this finding was not replicated in the third center that undertook DEXA screens. We proceeded to undertake micro-computed tomography (μCT) analysis of the two strains (Figure 4), and found that cortical thickness, cortical porosity, and trabecular bone volume were unchanged between N and J. In addition, analysis of various micro-architecture parameters indicated that the overall trabecular network was similar. Finally, measurement of bone formation and resorption markers failed to reveal any differences between the two strains (Figure 4).

#### Neurological, behavioral, and sensory

Two centers showed major and consistent differences between N and J in activity in the open field (ESLIM_007) (Figure 2), including higher activity in J mice as measured by distance travelled, and a higher number of center entries, indicative of reduced anxiety. These differences are in accord with data reported recently on a behavioral comparison of N and J [24]. Interestingly, the most significant effects were confined to males in the two centers. Unexpectedly, in a third center, the reverse was seen, with N mice being more active than J, although these effects were seen in both males and females. A fourth center did not detect these effects, finding no significant differences. The centers all used the EMPReSSslim SOP for the procedure, which included a requirement for similar-sized arenas, but there were some operational differences between the centers, including use of single or multiple rooms to house the arenas; transparent-sided or opaque-sided arenas; and the absence or presence of environmental enrichment in home cages (which is known to have an effect on behavioral outcomes [25]). However, none of these variables were consistent with the differing observations between centers. However, we cannot exclude influences of the gut microbiome, which might be expected to differ between centers. The gut microbiome is known to influence central nervous system function and behavior, mainly through the hypothalamic-pituitary-adrenal axis [26]. We conclude that under certain conditions, significant differences in open-field parameters between N and J can be seen, but the nature of these differences is sensitive to unknown environmental conditions. It is interesting that the major contradictory finding in the N versus J phenotypes was confined to a behavioral phenotyping platform. By contrast, for most other tests (aside from a few hematological and clinical chemistry parameters, see below), we did not find inconsistencies, indicating that in contrast to most phenotyping platforms, behavioral analyses can be acutely sensitive to environmental parameters.

**Figure 5:**
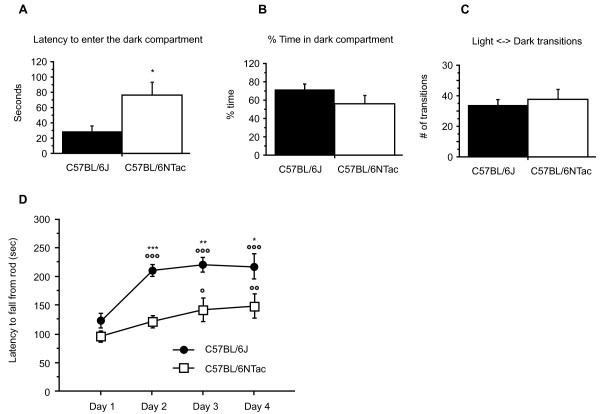
**Light/dark test**. Bars represent **(A) **the latency to enter, **(B) **the percentage of time spent in the dark compartment **(C) **and the number of light/dark transitions by C57BL/6J (*n *= 10) and C57BL/6N (*n *= 9) male mice, aged 8 to 10 weeks. Data are mean ± SEM, *P < 0.05 (t-test). **(D) **Rotarod motor learning performance over 4 days. Symbols and lines represent daily latencies (mean ± SEM) to fall from rotating rod at acceleration from 4 to 40 rpm in 300 seconds by C57BL/6J (*n *= 10) and C57BL/6N (*n *= 10) male mice, aged 9 to 11 weeks. *P < 0.05, **P < 0.005, ***P < 0.0001 J versus. N (*t*-test); °*P *< 0.05, °°*P *< 0.005, °°°*P *< 0.0001 versus day 1, Fisher's (least squares difference).

We also carried out a light/dark transition test to compare anxiety in N and J strains (Figure 5). We found no significant differences between N and J mice in the number of light-dark transitions or in the percentage time spent in the dark compartment. However, the latency to enter the dark compartment was significantly higher in N mice. Modified SHIRPA (SmithKline Beecham, Harwell, Imperial College, Royal London Hospital, Phenotype Assessment) testing (ESLIM_008, Figure 1a) in all four centers indicated that male J mice had significantly increased locomotor activity, which correlated with the findings of increased distance travelled in open-field testing in some centers (see above).

We conducted a number of tests that reflect motor ability. Differences in grip-strength (ESLIM_009) were seen across all centers with J being higher than N, but the parameters affected were different, with some centers reporting differences in forelimb grip-strength and some for forelimb and hindlimb grip-strength combined (Figure 1a, b). Rotarod testing (ESLIM_010) showed significant differences in latency to fall across all centers, although the reduced motor ability of N was only seen for females in two of the centers. We further explored motor abilities in N and J male mice by examining motor learning performance on the rotarod over 4 days (Figure 5). Whereas the motor performance of J mice improved markedly from day 1 to day 2, the performance of N mice improved only gradually, and was significantly different from the day 1 measurements only from day 3 (*P *< 0.05) onwards. Moreover, from day 2 to day 4 there were highly significant differences in the latency to fall between N and J. The primary testing carried out at the centers thus uncovered a potential reduced motor performance in N that was confirmed and further elaborated by more sophisticated testing of motor learning performance.

**Figure 6:**
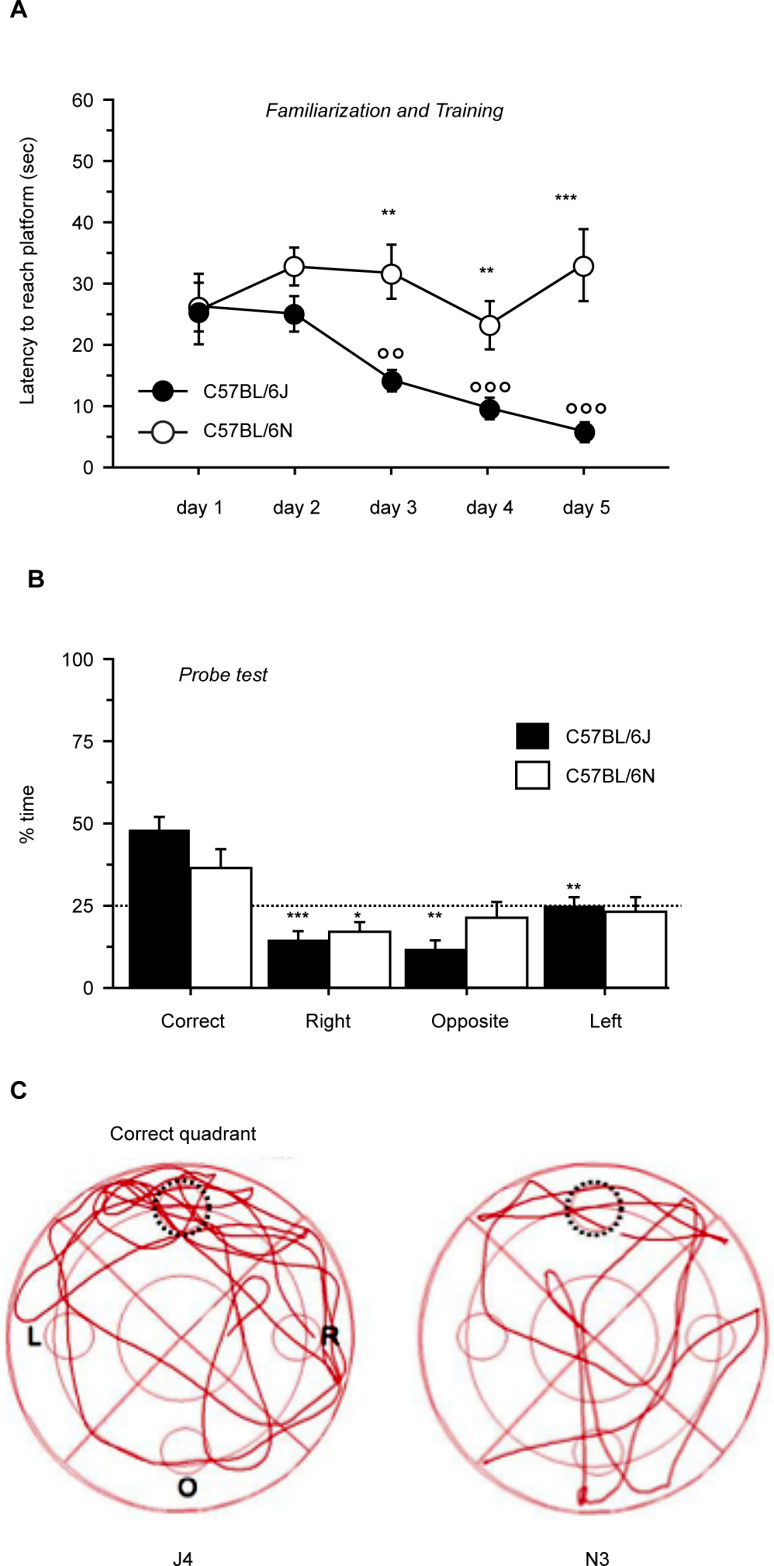
**Morris water maze**. **(A) **Learning curves for familiarization and training phases Symbols and lines represent daily latencies (mean ± SEM) to reach the platform by C57BL/6J (*n *= 10) and C57BL/6N (*n *= 10) male mice, aged 16 to 20 weeks. **P < 0.005, ***P < 0.0005 J versus N (*t*-test); °°*P *< 0.005, °°°*P *< 0.0001 versus day 1 (Fisher's least squares difference). **(B) **Probe test. Bars represent % time spent in each quadrant on day 5 during probe test. Dotted line is set at chance level (25%). *P < 0.05, **P < 0.005, ***P < 0.0005 versus correct quadrant (*t*-test)_. **(C) **Representative tracks of two C57BL/6J and C57BL/6N mice paths during probe test. Dotted circle indicates former platform location.

We also carried out two additional behavioral tests to further elaborate N versus J differences. Firstly, we compared the performance of N and J in the Morris water maze test used to assess spatial memory. N male mice showed very significantly reduced performance (higher latency) compared with J male mice (Figure 6). Secondly, we examined emotional learning or memory for an aversive event using the cue and contextual fear conditioning tests; however, here we found no significant differences between the two strains (data not shown).

Exploration of acoustic startle and pre-pulse inhibition (ESLIM_011) (Figure 1a) in the two strains identified a variety of parameters that were significantly and consistently different across centers. Acoustic startle magnitude at 110 dB and startle response magnitude to pre-pulse and pulse (PP1-PP4 + pulse, see EMPReSSslim) were reduced in N compared with J, although this effect was not seen in females in one center. Consistent with these observations we found that pre-pulse inhibition differed between N and J, with pre-pulse inhibition at PP2 and PP3 and global inhibition increased in N compared with J. Several other startle magnitude and pre-pulse inhibition parameters showed significant effects in one or two centers (Figure 1b; see Additional file 3, Figure S2b, f) but no differences were seen in other centers. The observations on startle magnitude were not confounded by differences in hearing as we assessed auditory thresholds in both J and N mice using the auditory brainstem response test and found no differences (data not shown).

#### Clinical chemistry

Extensive panels of clinical chemistry tests were performed on plasma samples collected at the end of each phenotyping pipeline. The blood sample at the end of Pipeline 1 (ESLIM_021) was collected after an overnight fast, whereas the sample at the end of Pipeline 2 (ESLIM_015) was from a free-fed animal. Data agreeing from at least three centers showed that urea and the electrolytes sodium, potassium, and chloride were significantly higher in plasma from J mice relative to N mice (Figure 1a), although there was some clear sex-center interactions. Data for free-fed and fasted plasma glucose levels indicated that for each test, at least two centers found plasma glucose levels to be higher in N than in J mice (Figure 1b). However, blood glucose levels are known to be affected by animal handling, sample processing, and the use of anesthetics. The data presented here are all from samples collected under gaseous isofluorane anesthetic, aside from one center in which samples were collected under ketamine/xylazine injection (see Figure 1). As discussed above, because of their known impairment in insulin secretion, it seems contradictory for J mice to have lower plasma glucose levels than N mice, but the deletion in *Nnt *appears to affect glucose clearance rates only, and fasted or non-challenged J mice do not have constant hyperglycemia. Several other parameters were shown to be higher in J than in N mice in at least two centers, but in each case the other center(s) reported no significant differences in the same parameters (Figure 1b), for example, free fatty acids. Two centers found that iron was significantly higher in N males and that alkaline phosphatase was significantly higher in J males. One of these centers also found the same to be true in females (Figure 2). However, data for each of these parameters from a third center contradict these findings.

#### Hematology

Various hematological parameters were measured at the end of Pipeline 2 (ESLIM_016). Significant changes in a number of parameters were found by two centers but these results were not replicated in the others, including white and red blood cell counts, mean cell volume, and mean corpuscular hemoglobin (Figure 1b). Contradictory results were obtained for hematocrit and mean cell hemoglobin concentration tests (Figure 2). In each case, data from two centers agreed whereas a third center showed the opposite effect. This could potentially be due to the different machine technologies used for hematological measurements in the participating clinics, as recorded in the metadata.

#### Immune function and allergy

**Figure 7:**
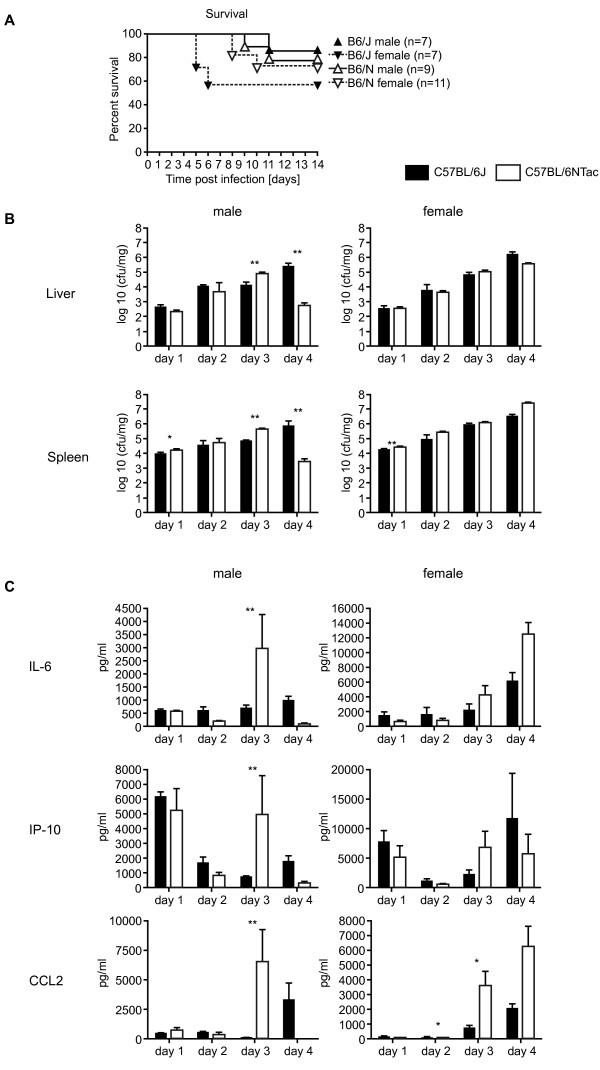
**Comparison of *Listeria *host resistance between C57BL/6J and C57BL/6N inbred strains**. **(A) **Kaplan-Meier survival curves of females and males of the C57BL/6J and C57BL/6N strains after intravenous (IV)v. infection with 2 × 10^4 ^colony-forming units (cfu) of *Listeria monocytogenes *strain EGD. **(B) **Bacterial load in liver and spleen of C57BL/6J and C57BL/6N mice after IV infection with 2 × 10^4 ^cfu *L. monocytogenes *EGD. Organ loads were ascertained at four time points to analyze kinetics of bacterial growth. **(C) **Comparison of plasma levels of interleukin (IL)-6, interferon-inducible protein (IP)-10, and chemokine ligand (CCL)2 between the C57BL/6J and C57BL/6N mice shown in **(B)**. Concentrations of pro-inflammatory cytokines and chemokines were determined in peripheral blood samples using the Cytokine Mouse 20-Plex Panel (Invitrogen Inc., Foster City, CA, USA) and a LiquiChip 100 system (Qiagen, Hilden, Germany). Significant differences are indicated as follows: *P < 0.05, **P < 0.01 Mann-Whitney, U-test. Black bars and symbols, C57BL/6J inbred strain. White bars and symbols, C57BL/6N inbred strain.

We investigated a number of secondary phenotypes including host resistance to *Listeria monocytogenes *in the J and N strains. Females of both strains were more susceptible to *L. monocytogenes *infection; however, the sex difference in *Listeria *host susceptibility was less pronounced in N than in J. Males of the N strain showed enhanced clearance of *Listeria *on day 4 post-infection compared with J males. This correlates with an increased pro-inflammatory response in N males on day 3 post-infection compared with J males (Figure 7).

**Figure 8:**
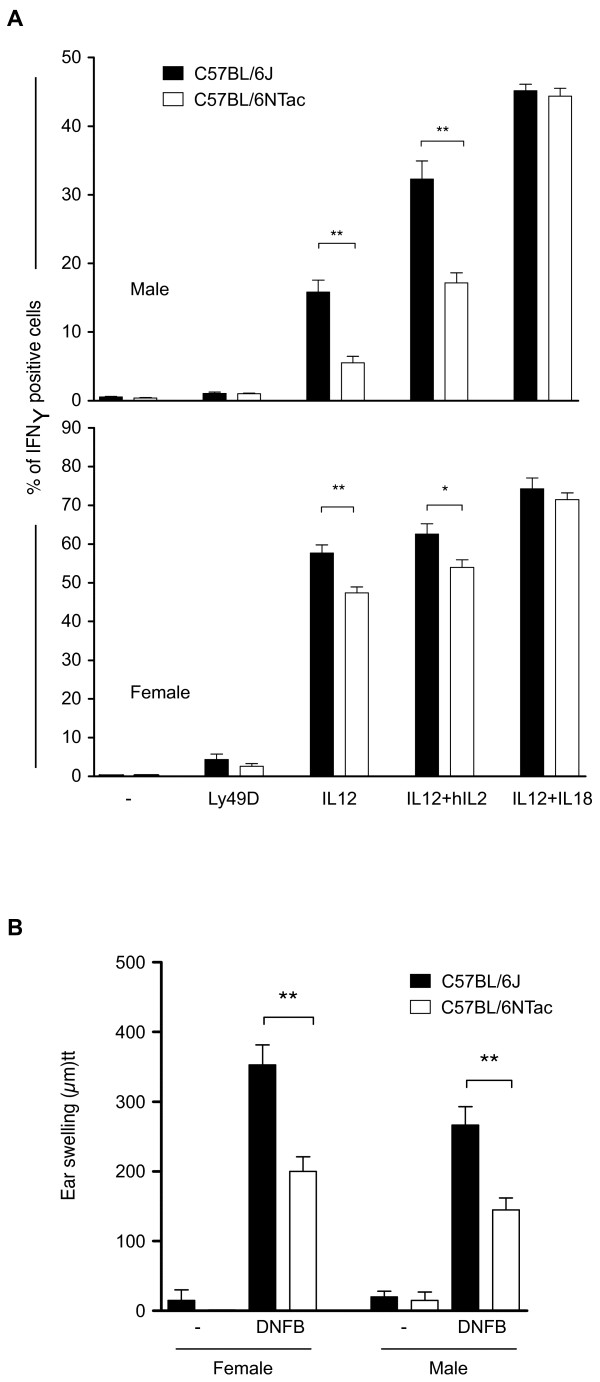
**Measurement of splenic natural killer (NK) cells and hapten-specific hypersensitivity**. **(A) **Splenic NK cell activity of C57BL/6J (B6J) versus C57BL/6N (B6NTac) mice: (upper panel) male and (lower panel) female. Splenic NK cells from C57BL/6J or C57BL/6N mice were stimulated under the indicated conditions (six mice per group). Mean ± SD of interferon (IFN)γ-positive cells among a population of CD3- NK1.1+ NK cells was measured by flow cytometry. **(B) **Hapten-specific hypersensitivity. Male or female C57BL/6J or C57BL/6N mice were sensitized by the application of 25 μl of 0.5% dinitrofluorobenzene (DNFB) solution on the ventral skin. They were then challenged by the application of 5 μl of 0.15% DNFB solution on the left ear 5 days later (DNFB group). The right ears were painted with vehicle (-) and used as controls. Ear thickness was measured 48 hours after challenge. Results are representative of three independent experiments with six mice per group. *P < 0.05; **P < 0.005 (Mann-Whitney *U*-test).

We also tested N and J mice for dinitrofluorobenzene (DNFB)-induced contact hypersensitivity (CHS). Significant differences in the CHS response were identified between the two strains of mice, with J showing an increased CHS response. Noticeably, female mice of both strains showed an increased CHS compared with male mice. Investigation of the responsiveness of natural killer (NK) cells to various stimuli showed that a larger fraction of NK cells are activated by interleukin (IL)-12 alone or in combination with IL-2 in J compared with N mice; again, this response was more significant in females (Figure 8).

## Discussion

We found that there are significant phenotypic differences between N and J mice covering a number of physiological, biochemical, and neurobehavioral systems. These findings have been replicated across a number of centers, indicating that the differences are robust to environmental variables and are likely to have an influence upon the comparative analysis of mutations in the two backgrounds in most laboratories. The phenotype differences we find between N and J will require careful consideration when comparing the effects of mutations created in the two genetic backgrounds. Although the use of C57BL/6N ESCs has allowed the rapid creation of a valuable genome-wide mutation resource [1], our findings of phenotypic differences between N and J indicates that the analysis of the phenotype data that will emerge from the IKMC resource will require careful interpretation in the context of the considerable legacy of data accumulated for C57BL/6J mutations.

The phenotypic differences between N and J can probably be accounted for, at least in part, by variations affecting coding sequences in the two genomes. We catalogued and validated a total of 36 SNPs and small indels affecting coding sequences along with a total of 43 SVs between the two genomes. In total, we identified 51 genes carrying some sequence variant or SV that might affect gene function. A proportion of these variants are likely to have little or no phenotypic consequence, including many missense mutations, and also SVs that do not overlap coding sequence. However, given the pleiotropic nature of most genetic loci and additive and epistatic effects, it seems likely that the catalogued coding variations will account for a considerable proportion of the differing phenotypes between the N and J strains.

We proceeded to test this proposition by comparing the phenotypes arising from knockouts of the identified loci with the phenotypes of N and J mice. First, we examined the available Mammalian Phenotype (MP) ontology terms, derived by analysis of mutant (usually knockout) phenotypes, for all the loci carrying an SNP, small indel, or SV between N and J. In so doing we attempted to draw correlations between phenotypes associated with individual variant loci and the phenotype changes seen between N and J. For many of the loci, as might be expected, no mutants have been characterized, and phenotypic annotations (MP terms) are not available. For loci carrying SNPs and small indels, 14 of the 36 loci had available MP terms. For SVs, where the SV overlaps a gene, we found MP terms for 7 out of 15 loci. In all cases, MP terms have been derived from knockout, presumably loss-of-function, mutations. For our analysis, we compared the loss-of-function phenotype found in the homozygous knockout with the phenotypic change between N and J, and identified candidate loci that might underlie the observed phenotype effects. For those loci for which we were able to make a comparison (see below), information on heterozygote phenotypes was not available, and therefore the analysis was confined to homozygotes.

There are number of assumptions inherent in this analysis. First, it is not trivial to consider the direction of phenotype effect that will arise from sequence variation between N and J. Although missense SNPs that are private to J or N may represent loss of function variants, this might not always be the case. SVs in the neighborhood of genes might also more often lead to loss-of-function effects, but other indirect effects, for example on gene regulation, might lead to gain of function. Second, knockout mutations have been generated on a variety of genetic backgrounds, often mixed, and this will confound any comparative analysis. Nevertheless, we proceeded to compare the available phenotype terms with the observed phenotype changes documented between N and J, assuming in the model that private variants in N or J are likely to lead to a loss-of-function phenotype of the kind that would be revealed by a knockout mutation.

Of the 14 SNP and small indel variant loci with phenotypic annotations, 5 (*Crb1*, *Pdzk1*, *Pmch*, *Adcy5*, *Nlrp12*) had MP terms that overlapped with the output of the phenotype tests undertaken by EUMODIC or by secondary phenotyping (Table 3). *Crb1 *carries the *rd8 *allele, a 1 bp deletion leading to a premature stop and a truncated protein. CRB1 is essential for external limiting membrane integrity and photoreceptor morphogenesis in the mammalian retina [27]. The *Crb1^rd8 ^*allele leads to shortened photoreceptor inner and outer segments 2 weeks after birth, and subsequent photoreceptor degeneration accompanied by regions of retinal hypopigmentation. We observed a very similar phenotype in the N mice, and in addition, using the optokinetic drum, we demonstrated significantly reduced vision in N compared with J mice. Moreover, we found that there were significant differences in the mean numbers of retinal veins and arteries between N and J mice. It has recently been reported that the *rd8 *allele is confined to the N sub-strain and derived ESCs [22]. For the locus *Pdzk1*, increased circulating cholesterol levels are reported in the knockout, but we did not observe this phenotype difference between N and J.

**Table 3:** Comparison of predicted effects of SNPs and SVs that might contribute to the phenotypic differences between C57BL/6N and C57BL/6J.^a^

Protein coding gene	C57BL/6J amino acid	C57BL/6N amino acid	SNP is private to:	PROVEAN prediction (score)^b^	MP terms	B6J versus B6N^2^	B6N versus B6J^c^
*Adcy5*	Valine (V)	Methionine (M)	B6N	Tolerated (-1.712)	Impaired coordination_MP:0001405	NR	P
					Hypoactivity_MP:0001402	NR	P
*Pmch*	Isoleucine (I)	Threonine (T)	B6N	Tolerated (0.493)	Decreased circulating glucose level_MP:0005560	NR	A
					Abnormal glucose tolerance_MP:0005291	NR	A
					Increased oxygen consumption_MP:0005289	NR	P
*Pdzk1*	Asparagine (N)	Aspartic Acid (D)	B6J	Tolerated (0.95)	Increased circulating cholesterol level_MP:0001556	A	NR
*Nlrp12*	Lysine (K)	Arginine (A)	B6J	Tolerated (0.781)	Abnormal type IV hypersensitivity reaction_MP:0002534	P	NR
*Crb1*	-	-	B6N	-	Photosensitivity_MP:0001999	NR	P
					Abnormal ocular fundus morphology_MP:0002864	NR	P
					Retinal degeneration_MP:0001326	NR	P
					Abnormal retinal morphology_MP:0001325	NR	P
					Abnormal retinal photoreceptor layer_MP:0003728	NR	P
*Chl1*	-	-	B6J	-	Abnormal learning/memory_ MP:0001449	A	NR
					Abnormal spatial working memory_MP:0008428	A	NR
*Rptor*	-	-	B6J	-	Increased lean body mass_MP:0003960	P	NR
					Increased oxygen consumption_MP:0005289	A	NR
					Hypoactivity_MP:0001402	A	NR
					Decreased circulating glucose level_MP:0005560	P	NR
					Improved glucose tolerance_MP:0005292	A	NR
*Nnt*	-	-	B6J	-	Impaired glucose tolerance_MP:0005293	P	NR

Abbreviations: A, phenotype absent; MP, Mammalian Phenotype; NP, Single-nucleotide polymorphism; NR, not relevant; P, phenotype present; PROVEAN, Protein Variation Effect Analyzer; SOP, Standard operating procedure; SV, structural variant.

^a^We have identified variant genes that show homozygote knockout phenotypes with associated MP terms that were assessed in the phenotyping pipeline, and compared these phenotypes to those seen between N and J.

^b^Threshold for intolerance is -2.3.

^c^These columns indicate the direction of the phenotype effect that might be seen given the assignment of a SNP or SV as private to B6J or B6N. Only one direction will be relevant and comparable with the effects of the knockout mutation.

For three of the loci (*Pmch*, *Adcy5*, *Nlrp12*) however, we did find some comparable phenotypic effects.

*Pmch *knockout mice display decreased circulating glucose, abnormal glucose tolerance, and increased oxygen consumption. N mice carry a private missense variant in this gene (isoleucine to threonine), and display increased oxygen consumption, but higher circulating glucose levels and normal glucose tolerance compared with J mice.

*Adcy5 *knockout mice display hypoactivity, impaired coordination, decreased vertical activity, and bradykinesia. N mice carry a private missense variant (valine to methionine) in *Adcy5*. N mice displayed a number of behavioral changes in open field, reflecting hypoactivity, including distance travelled and number of entries to the center. However, these phenotype outcomes were found by only two of the centers, with a third center finding opposing effects, and no changes being found in a fourth center (see above). Both primary and secondary phenotyping employing the rotarod identified significantly impaired motor coordination in N mice. For both missense variants in *Pmch *and *Adcy5*, the Protein Variation Effect Analyzer (PROVEAN) predictions indicated that the changes may not have a deleterious effect on protein function [28].

NLRP12 is known to be associated with auto-inflammatory disease in humans [29], and mutations in the NBS and NOD domains can cause periodic fever syndromes. *Nlrp12 *knockout mice show attenuated inflammatory responses for CHS [30]. J mice carry a private missense variant (arginine to lysine) in *Nlrp12 *that resides in a C-terminal leucine-rich repeat region of the gene. However, we found that J mice show an increased response to DNFB-induced CHS, suggesting that the *Nlpr12 *locus is not involved or, alternatively, that the missense mutation is a gain of function. Notably, most species (data not shown) carry a lysine at this position. PROVEAN predictions indicate that this mutation is not damaging to the protein.

For SVs with MP terms, three loci (*Chl1*, *Rptor*, *Nnt*) had MP terms that overlapped with the phenotype outputs generated in the EUMODIC pipeline (Table 3). *Chl1 *knockout mice demonstrate abnormal learning and memory, including abnormal response to a novel object and abnormal spatial working memory. *Chl1 *carries an intronic long interspersed element (LINE) insertion in J mice. However, N displayed impaired spatial working memory in the Morris water maze test compared with J mice, although it is worth noting that the poor performance of N mice could be explained by reduced vision that would impair their ability to decipher visual reference clues.

*Rptor *knockout mice demonstrate a large number of metabolic phenotypes including increased lean mass and reduced fat mass, improved glucose tolerance and decreased circulating glucose, increased oxygen consumption, and hypoactivity. *Rptor *carries an insertion (member of transcript retrotransposon (MTA)) in J mice, and these mice were found to have reduced fat mass and increased lean mass (in two of the centers), and decreased circulating glucose. However, a number of phenotypes that we have shown to be different in N and J mice are inconsistent with a loss-of-function mutation at the *Rptor *locus, including poor glucose tolerance in J mice, and increased oxygen consumption and hypoactivity in N mice.

Finally, J mice have been documented as carrying a large deletion at the *Nnt *locus [18], which is associated with significantly impaired glucose tolerance, and this phenotype was confirmed in our N versus J comparison. It is worth noting that given the expected strong effects of the *Nnt *locus on glucose tolerance, predicted effects from mutations at other loci on glucose tolerance may be over-ridden, and it is likely that *Nnt *will be epistatic to other loci. So for example, as we discuss above, whereas *Rptor *knockout mice showed improved glucose tolerance, we found that in J mice carrying an intronic MTA insertion in the *Rptor *gene, there is poor glucose tolerance. This may reflect the over-riding effect of the *Nnt *deletion on glucose regulation, or alternatively that the MTA insertion at *Rptor *has no effect on gene function.

## Conclusions

Functional analysis of the genetic mechanisms that underlie phenotypic traits in mouse mutants may be influenced, often profoundly, by genetic variation between individual inbred strains. In this study, we undertook the first analysis to detect and verify sequence variants between the two widely used mouse strains C57BL/6N and C57BL/6J. Using deep sequence data and comprehensive detection methods, we validated 51 coding variants, 34 coding SNPs, 2 indels and 15 SVs, differentiating C57BL/6N and C57BL/6J.

At the same time, we carried out a comprehensive phenotypic comparison of the two inbred strains and identified a considerable number of significant phenotype differences. While a direct analysis of the relationship between genomic variants and phenotypes were beyond the scope of this study, we thoroughly examined the landscape of phenotypic differences between the two strains, and where possible, related these to the known functions of the variant genes. The comparative examination of the phenotypic terms associated with knockout mutations and phenotype changes between N and J revealed some concordance and some discordance. These analyses were confounded by several factors, including the genetic background of the knockout mice and assumptions regarding the direction of phenotype effect of the variants discovered between N and J. In addition, for many SNPs and SVs, there may be little or no phenotype effect. However, our findings suggest a number of variants and loci that will merit further investigation by exploring the linkage between variant segregation and phenotype in N/J intercrosses. Moreover, N/J intercrosses would enable the identification of genetic loci underlying the many other phenotype differences between N and J, and allow us to explore the potential functional consequences of coding variation at the majority of loci for which there is as yet no functional annotation.

## Materials and methods

### Sequencing and genomic analyses

Full details on the mouse strains (C57BL/6J and C57BL/6N) used for sequencing and validation have been documented previously [11].

#### SNP and small indel identification

The paired-end alignment of C57BL/6N against the reference genome (C57BL/6J; also known as mm9/NCBIM37) [11] was used to find SNPs and small indels differentiating the two sub-strains. The raw sequence variant calls were made using GATK [12] with default parameters. We adopted a filtering strategy to reduce the number of false positives and lessen the burden for validation. SNP sites that occurred in the Broad J alignment, had an allele ratio of less than 0.8, or were located in a region of less than 3 or greater than 150 read depth were removed from further analysis. C57BL/6N BAM was realigned for calling small indels, and the above filtering procedure adopted. Annotation of the variants was performed with Annovar [14] and/or NGS-SNP [15]. Using these annotations, manual inspection of the coding variants (non-synonymous, splice donor-acceptor, or frameshift sites) removed sites embedded in homopolymers and GC-rich regions. The remaining coding variants and a subset of the non-coding variants were sent for Sequenom validation.

#### SNP and small indel validation

We designed extension and amplification primers for 762 SNPs and 169 small indels using SpectroDESIGNER, which were then synthesized (Metabion, Martinsried, Germany). We used the iPLEX GOLD assay of the Sequenom MassARRAY platform for genotyping these variants in eight DNA samples from three C57BL/6 sub-strains (replicate of four C57BL/6J and replicate of two C57BL/6NJ and C57Bl/6NTac) and SpectroTYPER for data analysis. The resulting genotypes were then downloaded and checked for consistency in the four replicates. Inconsistent or heterozygous genotypes in either the C57BL/6J or C57BL/6N samples were excluded from further analyses.

In addition to Sequenom, we used pyrosequencing and traditional Sanger sequencing for validation. We designed primers for 22 SNPs and 10 small indels. Primers were designed with Pyrosequencing™Assay Design, and oligonucleotides were synthesized at Eurofins MWG Operon. The PCR was performed using Taq Mastermix (Qiagen, West Sussex, UK). Samples were sent to GATC BioTech for sequencing, and pyrosequencing was carried out on the PSQ 96H Pyrosequencer. In cases with insufficient DNA or poor primer design, the SNPs or small indels were omitted from any further analysis.

C57BL/6N alignment files are available at the 17 Mouse Genomes Project [11]. Coding SNPs and indels are available from dbSNP [31,32].

#### ***SV identification***

Using a combination of four computational methods, as described previously [16], we detected a total of 551 genome-wide SVs between C57BL/6J and C57BL/6N. We then visually inspected short-read sequencing data at each of these 551 unique sites using LookSeq [33], and found that 470 predicted sites were false, owing to paired-end mapping errors. At the remaining 81 sites, we carried out PCR and Sanger-based sequencing analyses as described below.

#### SV validation

Primers were designed using Primer3 and purchased from MWG (Ebersberg, Germany). Primer design strategy was dependent on the type and size of the structural variant. Three independent PCR reactions were carried out with Hotstar Taq (Qiagen, Hilden, Germany). These reactions were performed as previously described [34]. A PCR kit (LongRange; Qiagen) was used for genomic regions greater than 2 kbp in length. PCR gel images were then assessed for quality of primer design and performance of PCR reaction. PCR products were then purified in a 96-well purification plate (Millipore), resuspended in 30 μl of water, and sequenced. All sequencing reactions were run out on an ABI3700 sequencer, and assembled using PHRED/PHRAP. PCR and Sanger-based sequencing analyses at the 81 retained sites allowed us to further remove 38 sites that were confirmed as not polymorphic between C57BL/6J and C57BL/6N and were due to reference errors. The structural variants are available from the Database of Genomic Variants Archive (accession ID: estd204) [35].

#### Predicted effects of sequence variants

Predicted effects of amino acid substitutions on their respective proteins were performed using PROVEAN [28]. MP ontology terms for genes containing SVs or SNPs was obtained from the Mouse Genome Database [36].

### Phenotyping

#### Phenotyping platforms in EMPReSSslim

All SOPs for phenotyping procedures are described within the EMPReSS database [37]. All mice were analyzed through the complete phenotyping pipeline (excluding FACS and immunoglobulin analyses, which were not undertaken). Subsequently subsets of mice were selected according to the appropriate metadata considerations to ensure robust comparisons between strains (see section on Phenotype data analysis below). Unless otherwise stated, C57BL/6NTac was used throughout the study. Secondary phenotyping protocols have been published previously [37].

#### Data capture by EuroPhenome

Data generated from EMPReSSslim by the four centers were stored in their local Laboratory Information Management Systems, backed by diverse database schemas running on different relational database management systems. The data were transferred to EuroPhenome in a common format Phenotype Data Markup Language, an extension of eXtensible Markup Language (XML), defined by XML schema. To assist in data export and improve standardization, and data consistency we provided a Java library [38] for data export. The informaticians at the centers used this to represent the data to be exported as an object model. The library then performed the necessary validation against the EMPReSS database [37]. If this was successful, the data were output to XML, compressed, and placed on a file transfer protocol (FTP) site.

Each center's FTP site was regularly checked by the EuroPhenome data capture system, and any new files were uploaded. The data were again verified against the schema and EMPReSS, and further checked for consistency against existing data within EuroPhenome. The results of the upload and validation were provided to the sites in the form of XML log files and a web interface, the EuroPhenome Tracker. If validation was successful the data were loaded into the EuroPhenome database. Data can be removed from the database by placing the files in the delete directory of the FTP site. The same process was used to capture and validate the data before removal. Phenotype data may be downloaded from MRC Harwell [39].

#### Phenotype data analysis

Statistical analysis was carried out in a manner consistent with the EuroPhenome data repository [19]. In order to compare phenotype data from N and J, groups of comparable measurements from each center were extracted from the EuroPhenome database. In a few instances there was more than one dataset from each center that differed according to a crucial metadata difference. Where there was more than one comparable group for a parameter, the largest one was used. An example was the simplified IPGTT procedure at the Helmholtz Institute, where the 'type of strip' was changed from 'accu-check aviva' to 'roche'. This resulted in two groups of data, one of sizes (20, 22) and one of (20, 13), for C57BL/6N and C57BL/6J respectively. The group with the largest minimum value within the two groups (in this case 20, 22), was used. Once comparable groups of C57BL/6J and C57BL/6N were identified, statistical tests were applied separately to the male and female groups for each center. A dataset of each group's sizes, means, standard deviations, effect sizes, and the resultant *P*-values from the statistical test was then created for subsequent hypothesis testing during the creation of the heat maps.

Fisher's exact test (for 2 × 2 contingency tables) or a χ^2 ^test were used for categorical data to produce a *P*-value. The Mann-Whitney *U*-test was applied to numerical data, as this is a non-parametric test suitable for all types of unimodal distributions. Two-dimensional data (such as parameters where a measurement is taken over a time course) were averaged into a single mean, which caused loss of information but still gave an overall comparable value for that parameter.

In order to create a multi-center heat map of statistical significances, a color and shade was selected for each parameter/site/sex combination, with a lower *P*-value (therefore indicating a higher confidence in the putative difference between the strains) resulting in a darker color. Where C57BL/6N values were greater than those of C57BL/6J, red was used, with green used for the opposite case. In categorical fields where there were no numerical values, blue was used to indicate a difference between the strains with no order.

In order to calculate the false-positive rate when significant results were found across multiple sites, a bootstrapping re-sampling technique [40] was used to estimate the probabilities of a parameter revealing similar trends in the same direction across three or more centers. All heat-map squares were randomized, and the number of times that three or more sites had squares of the same color was recorded. Repeated many times, and divided by the number of parameters and number of repeats, this provided a probability of that event occurring at random within the given heat-map. Comparison of this probability with the observed probability allowed us to assess whether we were looking at a purely random effect, or if there was underlying structure within the data. Analysis of the class of parameters with three or more centers showing trends in the same direction (Figure 1a) indicated that a randomized sample would show this in 0.074 of cases. This was compared with the observed rate of 0.213, indicating that this class was over-represented with respect to random chance. We applied the same analysis to the class of results that showed contradictory trends between centers (Figure 2). A randomized sample indicated that we would observe this pattern in 0.388 of cases, whereas the observed rate was 0.102, thus showing an under-representation, and again giving us confidence in these results. The class of results showing similar trends in two out of the four centers (Figure 1b) gave similar probabilities for random (0.183) and observed (0.181).

## Abbreviations

CCL: Chemokine ligand; cfu: Colony-forming units; CHS: Contact hypersensitivity; DNFB: dinitrofluorobenzene; DEXA: Dual energy X-ray absorptiometry; EMPReSS: European Mouse Phenotyping Resource of Standardised Screens; ESC: Embryonic stem cell; EUCOMM: European Conditional Mouse Mutagenesis project; EUMODIC: European Mouse Disease Clinic; FACS: Fluorescence-activated cell sorting; FTP: File transfer protocol; IFN: Interferon; IKMC: International Knockout Mouse Consortium; IL: interleukin; IMPC: International Mouse Phenotyping Consortium; IP: interferon-inducible protein; IPGTT: Intraperitoneal glucose tolerance test; IV: intravenous; KOMP: Knockout Mouse Project; LINE: Long interspersed element; μCT: micro-computed tomography (μCT); MP: Mammalian Phenotype; NK: natural killer; PROVEAN: Protein Variation Effect Analyzer; QTL: quantitative trait loci; SHIRPA: SmithKline Beecham, Harwell, Imperial College, Royal London Hospital, Phenotype Assessment; SNP: Single-nucleotide polymorphism; SOP: Standard operating procedure; SV: structural variant; VNTR: variable number tandem repeat; XML: eXtensible Markup Language.

## Competing interests

The authors declare that they have no competing interests.

## Authors' contributions

MMS, SG, JKW, HF, VG-D, SW, TS, KW, EB, EJC, SD, JE, JG, NJI, IJJ, AL, SM, JM, HMe, FP, OP, MR, PJ, BY, RR-S, KPS, A-MM, MHA, YH, and SDMB contributed equally, conceived and designed the experiments and wrote the paper. DJA, BP, JKW, HF, VG-D, TS, EB, EJC, SD, JE, JG, NJI, IJJ, AL, SM, JM, HMe, FP, OP, MR, PJ, KPS, YH, RD, SA, AA, LB, DB, HC, M-FC, RC, AF, A-KG, EG, WH, SMH, TH, WM, FN, L-AR, JR, MSa, MSe, CS, AS, MS, GT-V, VEV, SW, WW, and MZ performed the experiments. DJA, BP, MMS, SG, A-MM, SDMB, AB, PD, JMH, TMK, HMo, GN, LS, and HW performed statistical analysis and analyzed the data. All authors read and approved the final manuscript.

## Supplementary Material

Additional file 1 **Table S1, Single-nucleotide polymorphism (SNP) and small indel validation numbers**. Click here for file

Additional file 2 **Figure S1, The European Mouse Phenotyping Resource of Standardised Screens (EMPReSS)slim phenotyping pipeline**. **Figure S1. EMPReSSslim phenotyping pipeline**. The pipeline includes 20 phenotyping platforms. Data for FACS analysis of peripheral blood populations were not acquired for all centers and are not presented here. Click here for file

Additional file 3 **Figure S2 (a-h) Heat maps showing phenotyping parameter differences between the phenotyping centers**. **Figure S2. Heat maps and phenotype parameters. (A-D) **Heat maps (see Figure 1 and Figure 2) displayed with numbers of C57BL/6N (N) and C57BL/6J (J) animals analyzed for each test in each center. **(E-H) **Heat maps (see Figure 1 andFigure 2) showing the effect sizes seen in each test in each center. **(A, E) **Phenotype parameters that showed a significant difference between N and J in three or more centers. **(B, F) **Phenotype parameters that showed a significant difference between N and J in two centers but no evidence of trends in the other centers. **(C, G) **Phenotype parameters for which no significant differences were seen across the centers. **(D, H) **Phenotype parameters that showed significant differences in two or more centers, but the opposite trend in one of the centers. Click here for file
