# Supplementary material for: A comparative phenotypic and genomic analysis of C57BL/6J and C57BL/6N mouse strains
Source: Genome Biol. 2013 Jul 31;14(7):R82. doi: 10.1186/gb-2013-14-7-r82 (PMC4053787; doi:10.1186/gb-2013-14-7-r82)
Supplement: Additional file 1 — Table S1, Single-nucleotide polymorphism (SNP) and small indel validation numbers. [file gb-2013-14-7-r82-S1.PDF]

Table S1. SNP and small indel validation numbers

	**No. of filtered variants**	**No. variants sent for validation***	**Failed validation process****	**Loci not variant**	**No. validated*****
Coding SNPs	40	40	6	0	34
Non coding SNPs	8484	722	272	304	146
Coding indels	11	11	3	6	2
Non Coding indels	2142	158	82	22	54

* Validation is either by Sequenom, Pyrosequencing or Sanger Sequencing

** Variants failed the validation process when results were ambiguous due to a number of reasons including heterozygous genotypes, discrepancies between replicas and insufficient amplified DNA due to poor primer design.

*** Only variants with consistent genotypes where all replicas of B6N share the same genotype, all replicas of B6J share the same genotype, and the genotypes of B6N and B6J are different were considered validated.
