# Supplementary figures and images for: A comparative phenotypic and genomic analysis of C57BL/6J and C57BL/6N mouse strains
Source: Genome Biol. 2013 Jul 31;14(7):R82. doi: 10.1186/gb-2013-14-7-r82 (PMC4053787; doi:10.1186/gb-2013-14-7-r82)

# Pipeline 1

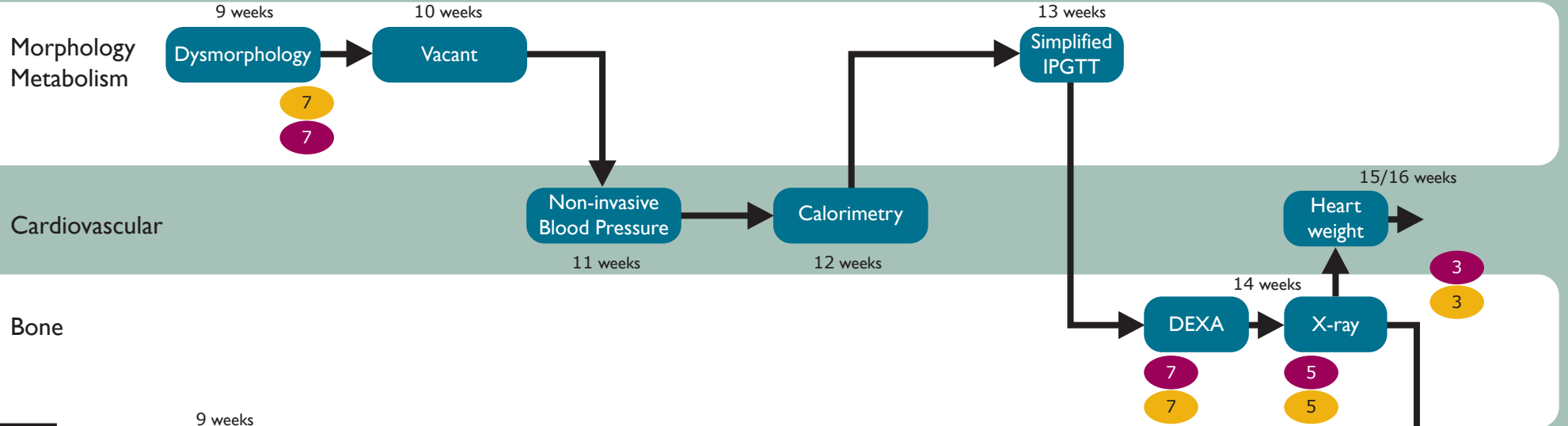

# Pipeline 2

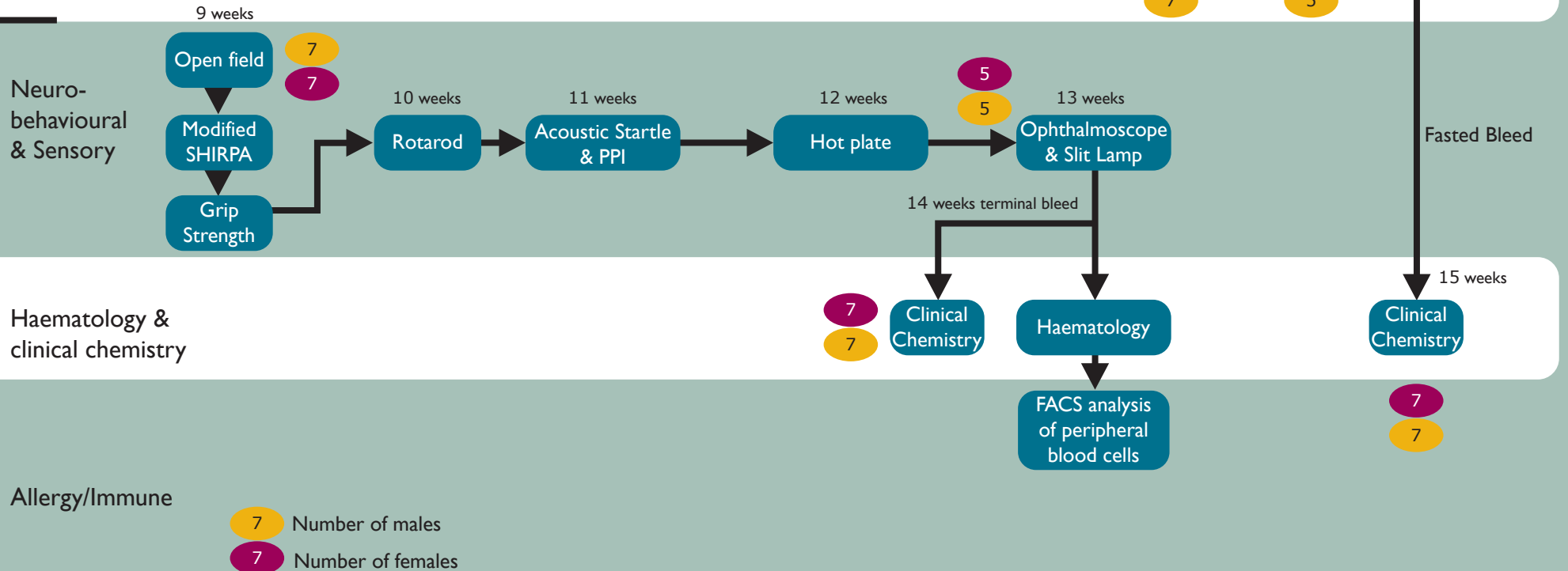

Supplement: Additional file 2 — Figure S1, The European Mouse Phenotyping Resource of Standardised Screens (EMPReSS)slim phenotyping pipeline. Figure S1. EMPReSSslim phenotyping pipeline. The pipeline includes 20 phenotyping platforms. Data for FACS analysis of peripheral blood populations were not acquired for all centers and are not presented here. [file gb-2013-14-7-r82-S2.PDF]
