# Supplementary material for: A comparative phenotypic and genomic analysis of C57BL/6J and C57BL/6N mouse strains
Source: Genome Biol. 2013 Jul 31;14(7):R82. doi: 10.1186/gb-2013-14-7-r82 (PMC4053787; doi:10.1186/gb-2013-14-7-r82)
Supplement: Additional file 3 — Figure S2 (a-h) Heat maps showing phenotyping parameter differences between the phenotyping centers. Figure S2. Heat maps and phenotype parameters. (A-D) Heat maps (see Figure 1 and Figure 2) displayed with numbers of C57BL/6N (N) and C57BL/6J (J) animals analyzed for each test in each center. (E-H) Heat maps (see Figure 1 andFigure 2) showing the effect sizes seen in each test in each center. (A, E) Phenotype parameters that showed a significant difference between N and J in three or more centers. (B, F) Phenotype parameters that showed a significant difference between N and J in two centers but no evidence of trends in the other centers. (C, G) Phenotype parameters for which no significant differences were seen across the centers. (D, H) Phenotype parameters that showed significant differences in two or more centers, but the opposite trend in one of the centers. [file gb-2013-14-7-r82-S3.PDF]

Parameter	Description	HMGU M	HMGU F	ICS M	ICS F	MRC Harwell M	MRC Harwell F	WTSI M	WTSI F
ESLIM_002_001_002	Non-Invasive blood pressure:Systolic arterial pressure	10.19	10.19	30.30	30.30	51.47	76.32	30.33	30.35
ESLIM_002_001_003	Non-Invasive blood pressure:Pulse rate	10.19	10.19	30.30	30.30	51.47	75.32	30.33	30.35
ESLIM_003_001_003	Calorimetry:Oxygen consumption	14.25	14.29	30.10	30.10	51.72	99.74	24.27	25.15
ESLIM_003_001_004	Calorimetry:Carbon dioxide production	14.25	14.29	30.10	30.10	51.72	99.74	30.27	28.15
ESLIM_003_001_006	Calorimetry:Heat production (metabolic rate)	14.25	14.29	30.10	30.10	51.72	99.75	30.27	28.15
ESLIM_004_001_002	Simplified IPGTT:Blood glucose concentration	20.21	19.22	29.28	30.30	101.17	96.18	30.33	30.30
ESLIM_004_001_701*	Simplified IPGTT:Glucose response AUC	20.21	19.22	29.28	30.30	101.17	96.18	30.33	30.30
ESLIM_005_001_002	DEXA:Fat mass	20.16	13.17	0.10	0.10	55.19	111.13	30.31	29.35
ESLIM_008_001_008	Modified SHIRPA:Locomotor activity	38.45	40.63	30.30	30.30	144.74	147.22	29.24	28.30
ESLIM_008_001_013	Modified SHIRPA:Startle response	38.45	40.63	30.30	30.30	144.74	144.22	29.24	28.30
ESLIM_009_001_001	Grip-Strength:Forelimb grip strength measurement	30.38	30.53	30.30	30.30	144.60	154.9	29.24	28.30
ESLIM_009_001_701*	Grip-Strength:Forelimb grip strength measurement mean	30.38	30.53	30.30	30.30	144.60	154.9	29.24	28.30
ESLIM_010_001_001	Rotarod: Latency to fall	38.45	40.63	30.30	30.30	139.92	132.29	29.24	28.30
ESLIM_010_001_002	Rotarod: Passive rotation	0.15	0.45	30.30	30.30	139.92	132.29	29.24	28.30
ESLIM_010_001_701*	Rotarod: Latency to fall mean	38.45	40.63	30.30	30.30	139.92	132.29	29.24	28.30
ESLIM_011_001_006	Acoustic Startle&PPI:110dB startle magnitude	38.30	40.38	29.30	29.30	86.64	117.19	29.5	28.10
ESLIM_011_001_007	Acoustic Startle&PPI:PP1 + pulse startle magnitude	38.30	40.38	29.30	29.30	86.64	117.19	29.5	28.10
ESLIM_011_001_008	Acoustic Startle&PPI:PP2 + pulse startle magnitude	38.30	40.38	29.30	29.30	86.64	117.19	29.5	28.10
ESLIM_011_001_009	Acoustic Startle&PPI:PP3 + pulse startle magnitude	38.30	40.38	29.30	29.30	86.64	117.19	29.5	28.10
ESLIM_011_001_010*	Acoustic Startle&PPI:PP4 + pulse startle magnitude	38.30	40.38	29.30	29.30	86.64	117.19	0.00	0.00
ESLIM_011_001_702*	Acoustic Startle&PPI:Prepulse inhibition - PP2	36.29	39.38	29.30	29.30	80.61	104.17	28.5	27.10
ESLIM_011_001_703*	Acoustic Startle&PPI:Prepulse inhibition - PP3	37.30	40.38	29.30	29.30	81.64	111.19	29.5	28.10
ESLIM_011_001_705*	Acoustic Startle&PPI:Global repulse inhibition	37.30	40.38	29.30	29.30	79.62	108.19	29.5	28.10
ESLIM_015_001_002	Clinical Chemistry:Urea	8.28	7.40	30.17	30.15	10.94	9.32	29.23	28.30
ESLIM_015_001_004	Clinical Chemistry:Sodium	8.28	7.40	30.17	30.18	10.94	9.32	29.24	28.28
ESLIM_015_001_005	Clinical Chemistry:Potassium	8.28	7.40	30.17	30.18	10.94	9.32	29.24	28.28
ESLIM_015_001_006	Clinical Chemistry:Chloride	8.28	7.40	30.17	30.18	10.94	9.32	29.24	28.28

**Key**	0.01	0.001	1.0E-4	1.0E-5	
N>J					No diff
N<J					No data available
N diff J					No data loaded

Figure S2A; Heat map (see Fig. 1a) displayed with numbers of C57BL/6N and C57BL/6J animals analysed for each test in each centre. Phenotype parameters that show a significant difference between N and J in 3 or more centres.

Parameter	Description	HMGU M	HMGU F	ICS M	ICS F	MRC Harwell M	MRC Harwell F	WTSI M	WTSI F
ESLIM_005_001_003	DEXA:Lean mass	20.16	20.17	0.10	20.0	55.19	111.13	30.31	29.35
ESLIM_005_001_004	DEXA:Bone Mineral Density (excluding skull)	20.16	20.17	20.0	20.0	55.19	111.13	30.31	29.35
ESLIM_007_001_007	Open-field:Periphery resting time	38.40	40.47	30.30	30.30	79.67	92.8	29.24	28.30
ESLIM_009_001_002	Grip-Strength:Forelimb and hindlimb grip strength measurement	30.38	30.53	30.30	30.30	144.60	154.9	29.24	28.30
ESLIM_009_001_702*	Grip-Strength:Forelimb and hindlimb grip strength measurement mean	30.38	30.53	30.30	30.30	144.60	154.9	29.24	28.30
ESLIM_011_001_001	Acoustic Startle&PPI:BN startle magnitude	38.30	40.38	29.30	29.30	86.64	117.19	29.5	28.10
ESLIM_011_001_002	Acoustic Startle&PPI:PP1 startle magnitude	38.30	40.38	29.30	29.30	86.64	117.19	29.5	28.10
ESLIM_011_001_701*	Acoustic Startle&PPI:Prepulse inhibition - PP1	35.30	39.36	22.20	26.23	46.34	59.12	29.5	28.10
ESLIM_011_001_704*	Acoustic Startle&PPI:Prepulse inhibition - PP4	38.30	40.38	29.30	29.30	81.63	112.19	29.5	28.10
ESLIM_012_001_002	Hot Plate:Type of response	20.25	20.35	30.30	30.30	102.20	119.19	29.24	28.30
ESLIM_015_001_007	Clinical Chemistry:Total protein			30.17	30.17	10.87	9.32	29.24	28.30
ESLIM_015_001_012	Clinical Chemistry:Lactate dehydrogenase			30.18	30.19	10.94	9.32	29.24	28.30
ESLIM_015_001_015	Clinical Chemistry:Alkaline phosphatase			0.64	0.28	1.43	0.91	1.60	1.24
ESLIM_015_001_016	Clinical Chemistry:Alpha-amylase			30.18	30.19	10.84	9.22	29.24	28.30
ESLIM_015_001_019*	Clinical Chemistry:Free fatty acid			0.9	0.7	18.0	19.0	29.24	28.29
ESLIM_016_001_001	Haematology:White blood cell count	38.38	39.53	27.29	30.29	9.101	22.31	29.24	28.30
ESLIM_016_001_002	Haematology:Red blood cell count	38.38	39.53	28.29	30.29	9.101	22.31	29.24	28.30
ESLIM_016_001_005	Haematology:Mean cell volume	38.38	39.53	28.29	30.29	9.101	23.31	29.24	28.30
ESLIM_016_001_006	Haematology:Mean corpuscular haemoglobin	38.38	39.53	28.29	30.29	9.99	23.31	29.24	28.30
ESLIM_020_001_002	Heart weight/tibia length:Heart weight	0.4	0.11	30.29	30.28	2.77	15.81	30.31	30.35
ESLIM_021_001_001	Fasted Clinical Chemistry:Glucose	0.0	0.0	20.29	20.30	10.36	10.59	30.31	30.34
ESLIM_021_001_003	Fasted Clinical Chemistry:Triglycerides	0.0	0.0	20.29	20.30	10.36	10.59	30.31	30.34
ESLIM_021_001_004	Fasted Clinical Chemistry:Free fatty acids	0.0	0.0	20.29	20.30	10.29	10.59	30.31	30.34

Key	0.01	0.001	1.0E-4	1.0E-5		
N>J					No diff	
N<J					No data available	
N diff J					No data loaded	

Figure S2B; Heat map (see Fig. 1b) displayed with numbers of C57BL/6N and C57BL/6J animals analysed for each test in each centre. Phenotype parameters that show a significant difference between N and J in 2 centres but no evidence of trends in the other centres.

Parameter	Description	HMGU M	HMGU F	ICS M	ICS F	MRC Harwell M	MRC Harwell F	WTSI M	WTSI F
ESLIM_003_001_007*	DEXA:Lean mass	0.0	0.0	30.10	30.10	0.0	0.0	30.27	28.15
ESLIM_003_001_008*	Calorimetry:Total activity (no. of fine movement + no. of beam cuts)	0.0	0.0	30.10	30.10	0.0	0.0	30.27	28.15
ESLIM_003_001_011*	Calorimetry:Total food intake	24.25	23.29	5.10	15.10	0.10	0.10	0.0	0.0
ESLIM_003_001_012*	Calorimetry:Cumulative food intake	0.0	0.0	30.10	30.10	0.0	0.0	30.23	26.15
ESLIM_003_001_701*	Calorimetry:Respiratory Exchange Ratio	14.25	14.29	30.10	30.10	51.72	100.75	0.0	0.0
ESLIM_005_001_005	DEXA:Bone Mineral Content	20.16	20.17	0.10	0.10	55.19	111.13	30.31	29.35
ESLIM_005_001_704*	DEXA:Bone area (BMC/BMD)	20.16	20.17	0.10	0.10	55.19	111.13	30.29	29.34
ESLIM_007_001_002	Open-field:Number of rears	38.40	40.47	30.30	30.30	0.0	0.0	29.24	28.30
ESLIM_007_001_003	Open-field:Whole arena resting time	38.40	40.47	30.30	30.30	79.67	92.8	29.24	28.30
ESLIM_007_001_004	Open-field:Whole arena permanence time	38.40	40.47	30.30	30.30	79.67	92.8	29.24	28.30
ESLIM_007_001_006	Open-field:Periphery distance travelled	38.40	40.47	30.30	30.30	79.67	92.8	29.24	28.30
ESLIM_007_001_012	Open-field:Centre distance travelled	38.40	40.47	30.30	30.30	79.67	92.8	29.24	28.30
ESLIM_007_001_013	Open-field:Centre average speed	38.40	40.47	30.30	30.30	79.67	92.8	29.24	28.30
ESLIM_007_001_014	Open-field:Latency to centre entry	38.40	40.47	30.30	30.30	79.67	92.8	29.24	28.30
ESLIM_007_001_702*	Open-field:Number of rears - total	38.40	40.47	30.30	30.30	0.0	0.0	29.24	28.30
ESLIM_008_001_001	Modified SHIRPA:Body position	38.45	40.63	30.30	30.30	144.74	147.22	29.24	28.30
ESLIM_008_001_002	Modified SHIRPA:Tremor	38.45	40.63	30.30	30.30	144.74	147.22	29.24	28.30
ESLIM_008_001_003	Modified SHIRPA:Defecation	38.45	40.63	30.30	30.30	144.74	147.22	29.24	28.30
ESLIM_008_001_004	Modified SHIRPA:Urination	38.45	40.63	30.30	30.30	84.74	102.22	29.24	28.30
ESLIM_008_001_005	Modified SHIRPA:Papebral closure	38.45	40.63	30.30	30.30	144.74	147.22	29.24	28.30
ESLIM_008_001_006	Modified SHIRPA:Lacrimation	38.45	40.63	30.30	30.30	144.74	147.22	29.24	28.30
ESLIM_008_001_007	Modified SHIRPA:Transfer arousal	38.45	40.63	30.30	30.30	144.74	147.22	29.24	28.30
ESLIM_008_001_009	Modified SHIRPA:Gait	38.45	40.63	30.30	30.30	144.74	147.22	29.24	28.30
ESLIM_008_001_011*	Modified SHIRPA:Pelvic elevation	20.28	20.43	10.0	10.0	84.74	102.22	29.24	28.30
ESLIM_008_001_012	Modified SHIRPA:Tail elevation	38.45	40.63	30.30	30.30	144.74	144.22	29.24	28.30
ESLIM_008_001_014	Modified SHIRPA:Touch escape	38.45	40.63	30.30	30.30	144.74	144.21	29.24	28.30
ESLIM_008_001_015	Modified SHIRPA:Positional passivity	38.45	40.63	30.30	30.30	144.74	144.22	29.24	28.30
ESLIM_008_001_016	Modified SHIRPA:Trunk curl	38.45	40.63	30.30	30.30	144.74	144.22	29.24	28.30
ESLIM_008_001_017	Modified SHIRPA:Limb grasping	38.45	40.63	30.30	30.30	144.74	144.22	29.24	28.30
ESLIM_008_001_018	Modified SHIRPA:Pinna reflex	38.45	40.63	30.30	30.30	144.74	144.22	29.24	28.30
ESLIM_008_001_019	Modified SHIRPA:Corneal reflex	38.45	40.63	30.30	30.30	144.74	144.22	29.24	28.30
ESLIM_008_001_020	Modified SHIRPA:Contact righting reflex	38.45	40.63	30.30	30.30	144.73	144.18	29.24	28.30
ESLIM_008_001_022	Modified SHIRPA:Vocalisation	38.45	40.63	30.30	30.30	144.74	144.22	29.24	28.30
ESLIM_011_001_003	Acoustic Startle&PPI:PP2 startle magnitude	38.30	40.38	29.30	29.30	86.64	117.19	29.5	28.10
ESLIM_011_001_004	Acoustic Startle&PPI:PP3 startle magnitude	38.30	40.38	29.30	29.30	86.64	117.19	29.5	28.10
ESLIM_011_001_005*	Acoustic Startle&PPI:PP4 startle magnitude	38.30	40.38	29.30	29.30	86.64	117.19	0.0	0.0
ESLIM_012_001_001	Hot plate:Time of first response	20.25	20.35	30.30	30.30	102.20	119.19	29.24	28.30
ESLIM_015_001_003	Clinical Chemistry:Creatinine			30.17	30.14	10.75	8.22	29.24	28.30
ESLIM_015_001_008	Clinical Chemistry:Albumin			30.18	30.19	10.94	9.32	29.24	28.30
ESLIM_015_001_009	Clinical Chemistry:Calcium			30.18	30.16	10.94	9.32	29.24	28.30
ESLIM_015_001_010	Clinical Chemistry:Phosphorus			30.18	30.18	10.94	9.32	29.24	28.30
ESLIM_015_001_013	Clinical Chemistry:Aspartate aminotransferase			30.17	30.18	10.94	9.32	29.24	28.30
ESLIM_015_001_014	Clinical Chemistry:Alanine aminotransferase			30.17	30.18	10.93	9.32	29.24	28.30
ESLIM_015_001_017	Clinical Chemistry:Total cholesterol			0.10	0.9	10.94	8.32	29.24	28.30
ESLIM_015_001_018	Clinical Chemistry:Triglyceride			0.10	0.9	10.94	8.32	29.24	28.30
ESLIM_015_001_020*	Clinical Chemistry:Creatine kinase			0.0	0.0	68.0	69.0	29.23	28.30
ESLIM_015_001_021*	Clinical Chemistry:Uric acid			0.0	0.0	0.0	0.0	29.23	28.30
ESLIM_015_001_022*	Clinical Chemistry:Total bilirubin			0.0	0.0	0.0	0.0	29.23	28.30
ESLIM_015_001_023*	Clinical Chemistry:HDL-cholesterol			0.0	0.0	0.0	0.0	29.24	28.30
ESLIM_015_001_024*	Clinical Chemistry:LDL-cholesterol			0.0	0.0	0.7	0.0	29.24	28.30
ESLIM_015_001_025*	Clinical Chemistry:Ferratin			0.0	0.0	0.0	0.0	0.0	0.0
ESLIM_015_001_026*	Clinical Chemistry:Transferrin			0.0	0.0	0.0	0.0	0.0	0.0
ESLIM_015_001_027*	Clinical Chemistry:C-reactive protein			0.0	0.0	0.0	0.0	0.0	0.0
ESLIM_016_001_003	Haematology:Haemoglobin	38.38	39.53	28.29	30.29	9.99	22.31	29.24	28.30
ESLIM_016_001_008	Haematology:Platelets count	38.38	39.53	28.29	30.29	9.100	22.31	29.24	28.30
ESLIM_020_001_003*	Heart weight/tibia length:Tibia length	0.0	0.0	30.29	30.28	0.0	0.0	0.0	0.0
ESLIM_020_001_004*	Heart weight/tibia length:Visual abnormality	0.4	0.14	30.29	30.28	2.18	15.29	30.31	30.35
ESLIM_020_001_006*	Heart weight/tibia length:Image	0.0	0.0	0.0	0.0	0.0	0.0	0.0	0.0
ESLIM_020_001_007*	Heart weight/tibia length:Histology abnormality	0.0	0.0	0.0	0.0	0.0	0.0	0.0	0.0
ESLIM_020_001_009*	Heart weight/tibia length:Histology image	0.0	0.0	0.0	0.0	0.0	0.0	0.0	0.0
ESLIM_021_001_002	Fasted Clinical Chemistry:Total cholesterol	0.0	0.0	20.29	20.30	10.36	10.59	30.31	30.34
ESLIM_021_001_005	Fasted Clinical Chemistry:HDL-cholesterol	0.0	0.0	20.29	20.30	10.36	10.59	30.31	30.34
ESLIM_021_001_006*	Fasted Clinical Chemistry:LDL-cholesterol	0.0	0.0	20.29	20.30	36.6	65.4	30.31	30.34
ESLIM_021_001_007*	Fasted Clinical Chemistry:Glycerol	0.0	0.0	20.29	20.30	10.29	10.49	30.31	30.34

Key	0.01	0.001	1.0E-4	1.0E-5		
N>J					No diff	
N<J					No data available	
N diff J					No data loaded	

Figure S2C; Heat map displayed with numbers of C57BL/6N and C57BL/6J animals analysed for each test in each centre. Phenotype parameters for which we did not observe any significant differences across the centres.

Parameter	Description	HMGU M	HMGU F	ICS M	ICS F	MRC Harwell M	MRC Harwell F	WTSI M	WTSI F
ESLIM_007_001_001	Open-field:Distance travelled	38.40	40.47	30.30	30.30	79.67	92.8	29.24	28.30
ESLIM_007_001_005	Open-field:Whole arena average speed	38.40	40.47	30.30	30.30	79.67	92.8	29.24	28.30
ESLIM_007_001_008	Open-field:Periphery permanence time	38.40	40.47	30.30	30.30	79.67	92.8	29.24	28.30
ESLIM_007_001_009	Open-field:Periphery average speed	38.40	40.47	30.30	30.30	79.67	92.8	29.24	28.30
ESLIM_007_001_010	Open-field:Centre distance travelled	38.40	40.47	30.30	30.30	79.67	92.8	29.24	28.30
ESLIM_007_001_011	Open-field:Centre resting time	38.40	40.47	30.30	30.30	79.67	92.8	29.24	28.30
ESLIM_007_001_015*	Open-field:Number of centre entries	38.40	40.47	30.30	30.30	79.67	92.8	29.24	28.30
ESLIM_007_001_701*	Open-field:Distance travelled - total	38.40	40.47	30.30	30.30	79.67	92.8	29.24	28.30
ESLIM_007_001_703*	Open-field:Percentage centre time	38.40	40.47	30.30	30.30	79.63	92.4	29.24	28.30
ESLIM_015_001_011	Clinical Chemistry:Iron			30.17	30.18	10.94	9.32	28.24	28.30
ESLIM_015_001_015	Clinical Chemistry:Alkaline phosphatase			30.17	30.18	10.94	9.32	29.24	28.30
ESLIM_016_001_004	Haematology:Haematocrit	38.38	39.53	28.29	30.29	9.101	22.31	29.24	28.30
ESLIM_016_001_007	Haematology:Mean cell haemoglobin concentration	38.38	39.53	28.29	30.29	9.99	23.31	29.24	28.30

Key	0.01	0.001	1.0E-4	1.0E-5	
N>J					No diff
N<J					No data available
N diff J					No data loaded

Figure S2D; Heat map (see Fig. 2) displayed with numbers of C57BL/6N and C57BL/6J animals analysed for each test in each centre. Phenotype parameters that showed significant differences in 2 or more centres, but the opposite trend in one of the centres.

Parameter	Description	HMGU M	HMGU F	ICS M	ICS F	MRC Harwell M	MRC Harwell F	WTSI M	WTSI F
ESLIM_002_001_002	Non-Invasive blood pressure:Systolic arterial pressure	1.94	0.80	1.44	1.34	0.68	0.38	1.25	1.68
ESLIM_002_001_003	Non-Invasive blood pressure:Pulse rate	0.30	-1.50	-1.12	-0.57	-1.35	-1.84	-3.28	-1.80
ESLIM_003_001_003	Calorimetry:Oxygen consumption	-1.08	-2.25	-0.53	-1.21	-0.33	-1.09	-0.28	-0.43
ESLIM_003_001_004	Calorimetry:Carbon dioxide production	-1.01	-2.50	-1.08	-1.50	-0.32	-1.04	0.18	0.02
ESLIM_003_001_006	Calorimetry:Heat production (metabolic rate)	-1.07	-2.35	-0.70	-1.34	-0.25	-1.33	-0.72	-0.65
ESLIM_004_001_002	Simplified IPGTT:Blood glucose concentration	0.86	1.80	0.59	1.09	1.11	1.03	0.95	0.71
ESLIM_004_001_701*	Simplified IPGTT:Glucose response AUC	1.11	1.56	0.87	1.30	1.14	1.28	1.13	0.56
ESLIM_005_001_002	DEXA:Fat mass	-1.54	-1.24			-2.35	-1.13	-2.27	-3.88
ESLIM_008_001_008	Modified SHIRPA:Locomotor activity	1.12	0.19	0.86	0.42	0.85	0.51	1.02	-0.24
ESLIM_008_001_013	Modified SHIRPA:Startle response								
ESLIM_009_001_001	Grip-Strength:Forelimb grip strength measurement	0.83	0.01	0.61	1.14	1.72	2.01	0.87	-0.08
ESLIM_009_001_701*	Grip-Strength:Forelimb grip strength measurement mean	0.83	0.01	0.61	1.14	1.72	2.01	0.87	-0.08
ESLIM_010_001_001	Rotarod:Latency to fall	0.67	0.54	1.23	1.32	0.73	-0.08	1.59	1.41
ESLIM_010_001_002	Rotarod:Passive rotation								
ESLIM_010_001_701*	Rotarod:Latency to fall mean	0.67	0.54	1.23	1.32	0.73	-0.08	1.59	1.41
ESLIM_011_001_006	Acoustic Startle&PPI:110dB startle magnitude	1.77	1.16	1.21	0.26	1.70	1.11	1.59	0.58
ESLIM_011_001_007	Acoustic Startle&PPI:PP1 + pulse startle magnitude	2.01	1.50	1.06	0.08	1.75	1.28	2.07	1.10
ESLIM_011_001_008	Acoustic Startle&PPI:PP2 + pulse startle magnitude	2.05	1.67	1.66	0.60	1.80	1.65	2.57	1.65
ESLIM_011_001_009	Acoustic Startle&PPI:PP3 + pulse startle magnitude	2.49	2.03	1.73	0.71	2.11	1.85	2.83	1.18
ESLIM_011_001_010*	Acoustic Startle&PPI:PP4 + pulse startle magnitude	2.40	2.31	1.25	0.74	1.83	1.94		
ESLIM_011_001_702*	Acoustic Startle&PPI:Prepulse inhibition - PP2	-0.87	-1.04	-1.16	-0.48	-0.34	-1.13	-2.59	-1.74
ESLIM_011_001_703*	Acoustic Startle&PPI:Prepulse inhibition - PP3	-1.37	-1.58	-0.93	-0.60	-0.57	-1.31	-2.62	-1.00
ESLIM_011_001_705*	Acoustic Startle&PPI:Global prepulse inhibition	-1.20	-1.31	-0.41	-0.18	-0.33	-0.97	-2.45	-1.39
ESLIM_015_001_002	Clinical Chemistry:Urea			1.61	1.23	0.51	0.18	0.94	0.53
ESLIM_015_001_004	Clinical Chemistry:Sodium			0.43	0.28	0.82	1.61	1.60	1.09
ESLIM_015_001_005	Clinical Chemistry:Potassium			1.93	1.56	0.26	2.28	0.43	-0.30
ESLIM_015_001_006	Clinical Chemistry:Chloride			0.56	0.04	1.36	2.15	2.13	1.78

**Key**	0.01	0.001	1.0E-4	1.0E-5		
N>J					No diff	
N<J					No data available	
N diff J					No data loaded	

Figure S2E; Heat map (see Fig. 1a) showing the effect sizes observed in each test in each centre. Phenotype parameters that show a significant difference between N and J in 3 or more centres.

Parameter	Description	HMGU M	HMGU F	ICS M	ICS F	MRC Harwell M	MRC Harwell F	WTSI M	WTSI F
ESLIM_005_001_003	DEXA:Lean mass	0.57	0.06			1.22	0.00	1.72	-0.54
ESLIM_005_001_004	DEXA:Bone Mineral Density (excluding skull)	-0.47	0.08			2.34	-0.35	1.51	0.00
ESLIM_007_001_007	Open-field:Periphery resting time	-0.29	-0.02	-0.77	-0.43	-0.21	0.79	-0.65	0.51
ESLIM_009_001_002	Grip-Strength:Forelimb grip strength measurement	1.34	0.85	0.28	0.19	0.96	2.31	0.28	-0.20
ESLIM_009_001_702*	Grip-Strength:Forelimb grip strength measurement mean	1.34	0.85	0.28	0.19	0.96	2.31	0.28	-0.20
ESLIM_011_001_001	Acoustic Startle&PPI:BN startle magnitude	-0.43	-0.89	-0.64	0.11	-0.01	-0.60	0.69	0.33
ESLIM_011_001_002	Acoustic Startle&PPI:PP1 startle magnitude	-0.61	-0.93	-0.45	0.01	-0.51	-1.22	0.10	-0.04
ESLIM_011_001_701*	Acoustic Startle&PPI:Prepulse inhibition - PP1	-0.79	-0.84	0.46	0.25	-0.49	-0.54	-1.32	-1.04
ESLIM_011_001_704*	Acoustic Startle&PPI:Prepulse inhibition - PP4	-1.32	-1.49	-0.05	-0.47	-0.18	-1.17		
ESLIM_012_001_002	Hot Plate:Type of response								
ESLIM_015_001_001	Clinical Chemistry:Glucose					-1.17	0.20	-0.31	-0.57
ESLIM_015_001_007	Clinical Chemistry:Total protein			0.77	0.62	-0.29	0.03	0.30	0.54
ESLIM_015_001_012	Clinical Chemistry:Lactate dehydrogenase			1.03	1.81	1.09	2.10	-0.32	0.43
ESLIM_015_001_016	Clinical Chemistry:Alpha-amylase			1.13	1.23	0.73	0.39	1.10	0.03
ESLIM_015_001_019*	Clinical Chemistry:Free fatty acid							-1.14	-1.06
ESLIM_016_001_001	Haematology:White blood cell count	0.62	0.30	0.63	1.07	-0.49	-0.78	0.36	-0.61
ESLIM_016_001_002	Haematology:Red blood cell count	-1.12	-1.24	0.10	0.56	0.51	0.62	-2.06	-0.95
ESLIM_016_001_005	Haematology:Mean cell volume	0.78	-0.02	0.41	-0.43	1.21	0.81	-0.11	-0.44
ESLIM_016_001_006	Haematology:Mean corpuscular haemoglobin	2.76	2.05	-0.22	0.01	-0.86	-0.37	2.41	1.71
ESLIM_020_001_002	Heart weight/tibia length:Heart weight			2.01	2.23	-0.38	0.39	1.30	-0.38
ESLIM_021_001_001	Fasted Clinical Chemistry:Glucose			-0.32	0.23	-2.94	-1.52	-1.88	-0.69
ESLIM_021_001_003	Fasted Clinical Chemistry:Triglycerides			0.95	0.69	1.54	0.76	0.35	0.05
ESLIM_021_001_004	Fasted Clinical Chemistry:Free fatty acids			0.92	-0.14	2.50	3.22	0.50	0.55

**Key**	0.01	0.001	1.0E-4	1.0E-5		
N>J					No diff	
N<J					No data available	
N diff J					No data loaded	

Figure S2F; Heat map (see Fig. 1b) showing the effect sizes observed in each test in each centre. Phenotype parameters that show a significant difference between N and J in 2 centres but no evidence of trends in the other centres.

Parameter	Description	HMGU M	HMGU F	ICS M	ICS F	MRC Harwell M	MRC Harwell F	WTSI M	WTSI F
ESLIM_003_001_007*	Calorimetry:Ambulatory activity (no. of beam cuts)			-0.92	-1.15			1.22	-0.33
ESLIM_003_001_008*	Calorimetry:Total activity (no. of fine movement + no. of beam cuts)			-0.92	-1.17			1.12	-0.40
ESLIM_003_001_011*	Calorimetry:Total food intake	-0.04	-0.67	0.12	0.41				
ESLIM_003_001_012*	Calorimetry:Cumulative food intake			-0.07	-0.10			-0.00	0.72
ESLIM_003_001_701*	Calorimetry:Respiratory Exchange Ratio	0.36	0.23	-1.23	-0.84	-0.09	-0.34		
ESLIM_005_001_005	DEXA:Bone Mineral Content	-0.53	-1.24			1.53	-0.27	0.59	-0.07
ESLIM_005_001_704*	DEXA:Bone area (BMC/BMD)	-0.27	-0.96			0.43	-0.16	-0.14	-0.67
ESLIM_007_001_002	Open-field:Number of rears	-0.35	0.09	0.00	-0.63			-0.14	-0.76
ESLIM_007_001_003	Open-field:Whole arena resting time	-0.55	-0.08	-0.53	-0.39	-0.90	-0.57	-0.42	0.80
ESLIM_007_001_004	Open-field:Whole arena permanence time					0.60	-0.03		
ESLIM_007_001_006	Open-field:Periphery distance travelled	-0.64	-0.82	0.17	-0.10	1.02	1.37	0.24	0.21
ESLIM_007_001_012	Open-field:Centre permanence time	-0.72	-0.45	2.32	1.20	0.35	-0.01	0.54	-0.22
ESLIM_007_001_013	Open-field:Centre average speed	-0.35	-0.61	0.05	0.14	1.00	1.23	-0.43	0.30
ESLIM_007_001_014	Open-field:Latency to centre entry	-0.21	0.06	-0.70	-0.15	-0.44	1.59	-0.03	-0.43
ESLIM_007_001_702*	Open-field:Number of rears - total	-0.35	0.09	0.00	-0.63			-0.14	-0.76
ESLIM_008_001_001	Modified SHIRPA:Body position								
ESLIM_008_001_002	Modified SHIRPA:Tremor								
ESLIM_008_001_003	Modified SHIRPA:Defecation								
ESLIM_008_001_004	Modified SHIRPA:Urination								
ESLIM_008_001_005	Modified SHIRPA:Papebral closure								
ESLIM_008_001_006	Modified SHIRPA:Lacrimation								
ESLIM_008_001_007	Modified SHIRPA:Transfer arousal								
ESLIM_008_001_009	Modified SHIRPA:Gait								
ESLIM_008_001_011*	Modified SHIRPA:Pelvic elevation								
ESLIM_008_001_012	Modified SHIRPA:Tail elevation								
ESLIM_008_001_014	Modified SHIRPA:Touch escape								
ESLIM_008_001_015	Modified SHIRPA:Positional passivity								
ESLIM_008_001_016	Modified SHIRPA:Trunk curl								
ESLIM_008_001_017	Modified SHIRPA:Limb grasping								
ESLIM_008_001_018	Modified SHIRPA:Pinna reflex								
ESLIM_008_001_019	Modified SHIRPA:Corneal reflex								
ESLIM_008_001_020	Modified SHIRPA:Contact righting reflex								
ESLIM_008_001_022	Modified SHIRPA:Vocalisation								
ESLIM_011_001_003	Acoustic Startle&PPI:PP2 startle magnitude	-1.10	-1.18	-0.25	0.75	-0.56	0.22	0.33	-0.22
ESLIM_011_001_004	Acoustic Startle&PPI:PP3 startle magnitude	-1.09	-1.01	0.48	0.75	-0.50	-0.03	0.40	-0.05
ESLIM_011_001_005*	Acoustic Startle&PPI:PP4 startle magnitude	-0.35	-0.53	0.67	0.33	-0.45	0.18		
ESLIM_012_001_001	Hot plate:Time of first response	-0.24	0.62	-0.72	-0.86	0.69	0.35	0.01	0.43
ESLIM_015_001_001	Clinical Chemistry:Glucose			0.10	0.9	10.94	9.32	29.24	28.30
ESLIM_015_001_003	Clinical Chemistry:Creatinine			0.19	0.83	0.05	0.13	-0.20	-1.01
ESLIM_015_001_008	Clinical Chemistry:Albumin			-0.11	-0.40	1.01	1.76	-0.32	0.43
ESLIM_015_001_009	Clinical Chemistry:Calcium			0.69	0.71	-1.03	-1.53	-0.25	1.20
ESLIM_015_001_010	Clinical Chemistry:Phosphorus			0.63	0.67	0.24	-0.46	0.88	-0.50
ESLIM_015_001_013	Clinical Chemistry:Aspartate aminotransferase			0.60	1.48	0.10	0.62	0.27	0.32
ESLIM_015_001_014	Clinical Chemistry:Alanine aminotransferase			0.64	1.61	-0.25	-0.62	-0.60	-0.66
ESLIM_015_001_017	Clinical Chemistry:Total cholesterol					0.25	0.50	0.07	-1.15
ESLIM_015_001_018	Clinical Chemistry:Triglyceride					-0.58	0.32	0.03	-1.31
ESLIM_015_001_020*	Clinical Chemistry:Creatine kinase							1.06	0.64
ESLIM_015_001_021*	Clinical Chemistry:Uric acid							1.22	-0.19
ESLIM_015_001_022*	Clinical Chemistry:Total bilirubin							1.24	1.20
ESLIM_015_001_023*	Clinical Chemistry:HDL-cholesterol							-0.08	-0.61
ESLIM_015_001_024*	Clinical Chemistry:LDL-cholesterol							-0.09	-1.43
ESLIM_015_001_025*	Clinical Chemistry:Ferratin								
ESLIM_015_001_026*	Clinical Chemistry:Transferrin								
ESLIM_015_001_027*	Clinical Chemistry:C-reactive protein								
ESLIM_016_001_003	Haematology:Haemoglobin	-0.46	-0.63	0.04	0.58	-0.22	0.49	-0.88	-0.28
ESLIM_016_001_008	Haematology:Platelets count	-0.49	-0.11	-0.14	-0.39	-0.59	-0.17	-0.15	-0.87
ESLIM_020_001_003*	Heart weight/tibia length:Tibia length			2.84	2.44				
ESLIM_020_001_004*	Heart weight/tibia length:Visual abnormality								
ESLIM_020_001_006*	Heart weight/tibia length:Image								
ESLIM_020_001_007*	Heart weight/tibia length:Histology abnormality								
ESLIM_020_001_009*	Heart weight/tibia length:Histology image								
ESLIM_021_001_002	Fasted Clinical Chemistry:Total cholesterol			0.14	-0.34	1.10	0.09	-1.93	-2.90
ESLIM_021_001_005	Fasted Clinical Chemistry:HDL-cholesterol			0.66	-0.20	1.17	0.42	-2.36	-3.18
ESLIM_021_001_006*	Fasted Clinical Chemistry:LDL-cholesterol			0.41	-0.07	-1.04	0.97	-2.47	-1.94
ESLIM_021_001_007*	Fasted Clinical Chemistry:Glycerol			0.62	0.97	-1.26	1.22	0.39	0.42

Key	0.01	0.001	1.0E-4	1.0E-5		
N>J					No diff	
N<J					No data available	
N diff J					No data loaded	

Figure S2G; Heat map showing the effect sizes observed in each test in each centre. Phenotype parameters for which we did not observe any significant differences across the centres.

Parameter	Description	HMGU M	HMGU F	ICS M	ICS F	MRC Harwell M	MRC Harwell F	WTSI M	WTSI F
ESLIM_007_001_001	Open-field:Distance travelled	-1.04	-1.07	1.00	0.56	1.06	1.05	0.39	-0.03
ESLIM_007_001_005	Open-field:Whole arena average time	-1.20	-0.99	1.24	0.51	1.06	1.07	0.24	0.35
ESLIM_007_001_008	Open-field:Periphery permanence time	0.72	0.45	-2.32	-1.20	0.43	1.13	-0.54	0.22
ESLIM_007_001_009	Open-field:Periphery average speed	-1.16	-0.93	0.99	0.25	1.13	1.37	0.36	0.44
ESLIM_007_001_010	Open-field:Centre distance travelled	-1.03	-0.87	2.39	1.44	0.75	0.74	0.35	-0.31
ESLIM_007_001_011	Open-field:Centre resting time	-0.84	-0.22	1.41	0.44	0.14	1.68	0.72	0.59
ESLIM_007_001_015*	Open-field:Number of centre entries	-1.34	-1.12	2.32	1.41	0.67	0.52	0.37	-0.42
ESLIM_007_001_701*	Open-field:Distance travelled - total	-1.04	-1.07	1.00	0.56	1.06	1.02	0.39	-0.03
ESLIM_007_001_703*	Open-field:Periphery centre time	-0.72	-0.45	2.32	1.20	-0.05	-2.79	0.54	-0.22
ESLIM_015_001_011	Clinical Chemistry:Iron			0.14	0.98	-1.64	-0.48	-1.10	-1.14
ESLIM_016_001_004	Haematology:Haematocrit	-0.70	-1.06	0.19	0.40	-1.29	1.11	-1.71	-0.95
ESLIM_016_001_007	Haematology:Mean cell haemoglobin concentration	0.69	1.16	-0.69	0.31	-1.72	-0.76	1.28	1.24

**Key**	0.01	0.001	1.0E-4	1.0E-5		
N>J					No diff	
N<J					No data available	
N diff J					No data loaded	

Figure S2H; Heat map (see Fig. 2) showing the effect sizes observed in each test in each centre. Phenotype parameters that showed significant differences in 2 or more centres, but the opposite trend in one of the centres.
